# Single pulse electrical stimulation in white matter modulates iEEG visual responses in human early visual cortex

**DOI:** 10.1371/journal.pcbi.1014563

**Published:** 2026-07-24

**Authors:** Harvey Huang, Kendrick N. Kay, Nicholas M. Gregg, Gabriela Ojeda Valencia, Myung-Ho In, Christoph Kapeller, Yunhong Shu, Gregory A. Worrell, Kai J. Miller, Dora Hermes

**Affiliations:** 1 Medical Scientist Training Program, Mayo Clinic, Rochester, Minnesota, United States of America; 2 Center for Magnetic Resonance Research, Department of Radiology, University of Minnesota, Minneapolis, United States of America; 3 Department of Neurology, Mayo Clinic, Rochester, Minnesota, United States of America; 4 Department of Physiology and Biomedical Engineering, Mayo Clinic, Rochester, Minnesota, United States of America; 5 Department of Radiology, Mayo Clinic, Rochester, Minnesota, United States of America; 6 Invasive Technologies, g.tec medical engineering GmbH, Schiedlberg, Austria; 7 Department of Neurologic Surgery, Mayo Clinic, Rochester, Minnesota, United States of America; Université Paris Cité: Universite Paris Cite, FRANCE

## Abstract

**Introduction:**

Electrical stimulation is increasingly used to modulate brain networks for clinical purposes. The basic unit of neurostimulation, a single electrical pulse, can travel through white matter to influence connected neuronal populations. However, the mechanisms by which it influences connected populations is not well understood: stimulation may excite, inhibit, or add noise to neuronal population activity.

**Materials and methods:**

In this study, we investigated how single pulses modulate the neuronal processing of images in a well-controlled visual paradigm. In two human subjects implanted with iEEG electrodes for clinical purposes, single pulses were delivered to electrodes in white matter tracts connected to measurement electrodes in visual cortex. Images appeared on-screen at 0, 100, or 200 ms after each pulse. Using finite impulse response modeling, we decomposed the broadband and evoked potential responses into separate components induced by electrical stimulation and by visual processing.

**Results:**

Single pulses induced transient broadband increases followed by suppression, but they did not modulate the visual broadband responses (i.e., stimulation response was additive to visual response). In contrast, single pulses elicited prominent brain stimulation evoked potentials *and* they modulated the visual evoked potentials. Specifically, visual evoked potentials were larger when stimulation occurred closer to visual onset. This indicates that a single electrical pulse can increase the strength or synchrony of visual inputs.

**Conclusion:**

Overall, these findings suggest that the effects of electrical stimulation in the visual system are two-fold: stimulation induces additive effects on broadband power, possibly by adding noise, and it interacts with synchronous visual inputs to amplify them.

## Author summary

Intracranial electrical stimulation treats various neuropsychiatric conditions including epilepsy, movement disorders, and mood disorders, through both approved and emerging therapies. However, therapeutic innovation remains limited by our incomplete understanding of how electrical stimulation interacts with brain function.

To elucidate this interaction, we borrowed linear modeling techniques from functional MRI research and applied them to human intracranial measurements. These techniques isolate the neuronal responses to visual processing, and they quantify how electrical stimulation modifies those responses. Our analysis characterizes how the basic unit of brain stimulation – a single electrical pulse – can travel through white matter tracts to influence neuronal populations that are engaged with the environment.

Our findings show that stimulation modulates visual processing by enhancing synchronous neuronal responses to visual inputs. This modulation occurred in a manner potentially consistent with added neuronal noise. Overall, our experimental paradigm and analysis framework effectively quantified the complex interactions between electrical stimulation and active neuronal processing, expanding beyond traditional approaches that examine stimulation effects on the resting brain alone.

## 1. Introduction

Intracranial neurostimulation has shown great promise as a treatment for a variety of neurological conditions. Current FDA-approved intracranial neurostimulation therapies reduce symptom burden for patients with movement disorders and medication-refractory epilepsy [1–3]. Emerging intracranial neurostimulation efforts also exist to restore vision [4–6] and treat psychiatric disorders such as depression and obsessive-compulsive disorder [7–9]. Many such applications target well-connected brain nuclei (e.g., thalamus) or white matter, and are thought to function by affecting connected areas within brain networks [10]. Despite clinical success, the mechanisms by which neurostimulation modulates natural dynamics within brain networks remain the subject of ongoing investigation. Some deep brain stimulation studies have posited that stimulation virtually lesions neuronal populations [10,11], while another theory based largely in transcranial magnetic stimulation and peripheral nerve stimulation literature suggests that stimulation injects neuronal noise [12,13]. Virtual lesions would be expected to suppress ongoing neuronal processing, whereas injected neuronal noise might result in added neuronal activity or nonlinear effects such as stochastic resonance [14,15]. These theories can be tested by carefully characterizing the interaction between electrical stimulation and neuronal activity.

A single electrical pulse is the basic unit of most neurostimulation therapies and is sufficient, in itself, to produce dynamic effects on local neuronal activity at connected measurement sites [16]. For example, single pulse electrical stimulation (SPES) through microelectrodes in macaque thalamus can elicit a transient increase followed by a sustained decrease in neuronal firing in connected visual cortex [17]. This resembles our previous population-level intracranial EEG (iEEG) findings, where single electrical pulses delivered to the hippocampus induced transient increases followed by sustained decreases in broadband power in the ventral temporal cortex [18]. These broadband power changes are thought to correlate with neuronal population firing rates [19–21] and represent a stimulation effect on population activity.

Whether SPES modulates neuronal activity not just at rest but also during active engagement remains a gap in understanding. We address this in the present study by combining iEEG SPES with a visual task in two human participants. We stimulated major white matter tracts connected to early visual areas (V1-V3) while presenting a visual stimulus at three different intervals after electrical stimulation. We separated the iEEG responses into stimulation-driven and visual-driven components and tested whether the visual components differed by stimulation condition. If so, this was taken as evidence that SPES modulates visual processing. We quantified stimulation-visual interactions for both broadband power changes and evoked potentials, which are two independent iEEG signal features that reflect complementary neurophysiological events [22,23]. Broadband power is related to asynchronous population firing [19–21], while evoked potentials quantify synchronous synaptic inputs [24,25]. Interpreting stimulation effects on broadband power and evoked potentials together helps us advance toward a comprehensive picture of how electrical pulses interact with dynamic neuronal activity.

## 2. Materials and methods

### 2.1. Ethics statement

The experiments in this study were conducted according to the guidelines of the Declaration of Helsinki and approved by the Institutional Review Board of the Mayo Clinic (IRB #15-006530), which also authorizes sharing of the deidentified data. Each patient in this study voluntarily provided independent written informed consent to participate in this study.

### 2.2. Subjects and iEEG recording

iEEG data were recorded in 2 human subjects (subject 1: 21M, subject 2: 18M) who had been implanted with stereo-EEG electrodes for epilepsy seizure localization. In each subject, iEEG electrodes were placed in the visual cortex and in other brain areas (subject 1: 192 electrodes, subject 2: 238 electrodes), as dictated by the clinical team. Recorded data were digitized at 4800 Hz on a g.Hiamp biosignal amplifier (g.tec medical engineering GmbH., Austria), and originally referenced to an electrode in the white matter.

### 2.3. Task and stimuli

**Fig 1:**
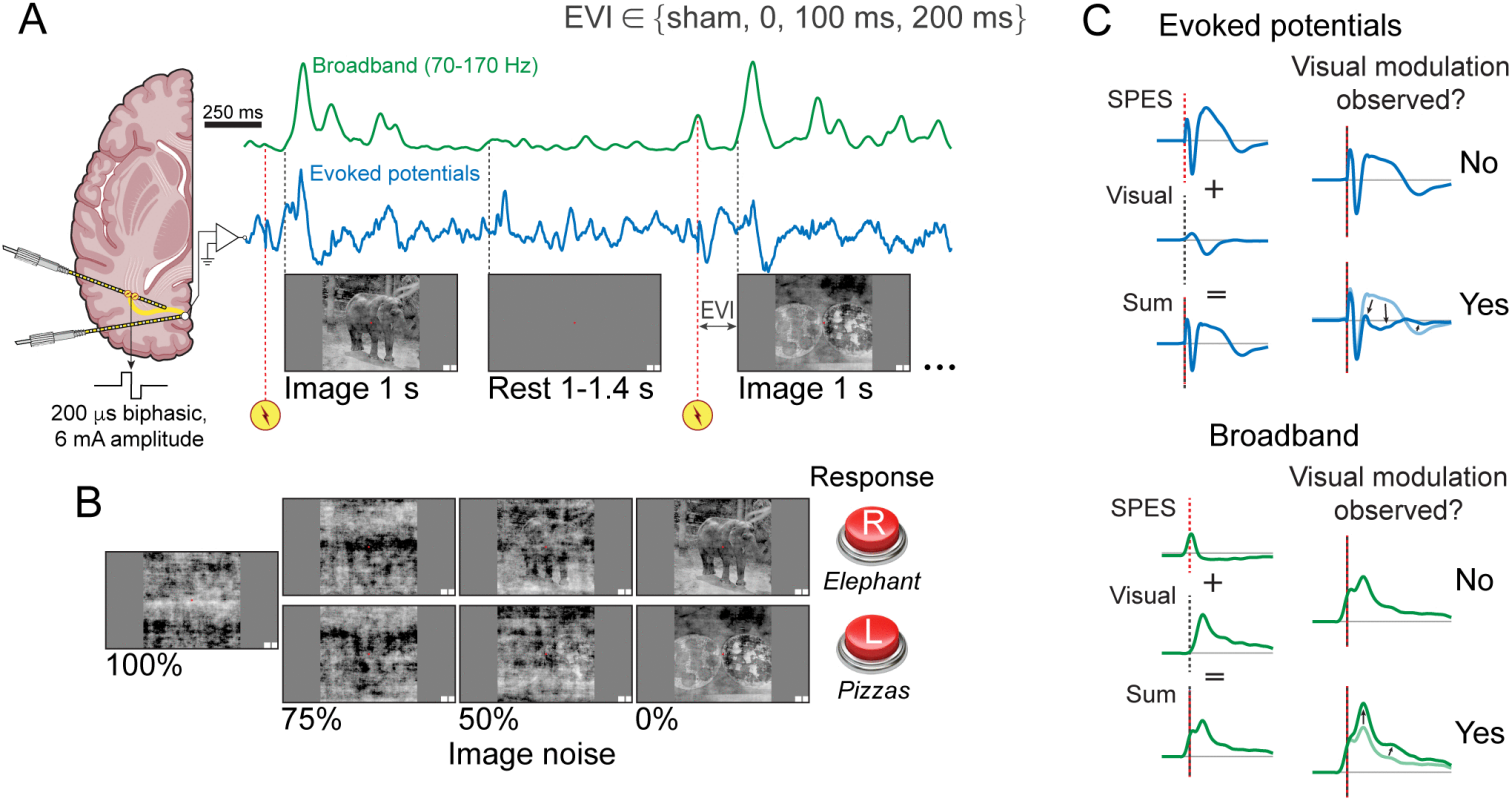
SPES+Visual experimental task and objectives. **A,** Subjects fixated on images of 1 s duration with 1-1.4 s rest, and SPES occurred in non-sham trials at 0, 100, or 200 ms before visual onset (EVI). Evoked potential time series were recorded and transformed to time-varying broadband estimates. Brain graphic created in part with BioRender.com. **B,** Seven image conditions corresponded to two natural scenes mixed with four levels of noise. Subjects were instructed to identify the natural scene by button press. Grayscale images in A and B are derivatives of natural scenes from the Common Objects in Context database (https://cocodataset.org, [26]). **C,** Schematic depicting the primary objective: to determine whether visual evoked potentials and visual broadband changes were modulated by SPES.

The main experiment combined SPES with a visual perceptual decision-making task (referred to as SPES+Visual). During the task, subjects fixated at the center of the screen, spanning ~28 degrees of visual angle at ~60 cm distance. Grayscale images constructed from two natural scenes were presented for 1 s each with 1-1.4 s of rest (uniform gray screen) in between (Fig 1A), via custom g.HIsys Simulink software (g.tec). Subjects were instructed to press a button to indicate which of two natural scenes was reflected in each image presented. Subject 1 also received short auditory feedback upon each button press to indicate response correctness (a “coin jingle” when correct, a buzz when incorrect).

The images were produced by modifying the phase spectra of two natural scenes, *Elephant* and *Pizzas*, according to the procedure in Heekeren et al. (Fig 1B) [27]. The two natural scenes were taken originally from the Common Objects in Context database (https://cocodataset.org, [26]). This procedure generated seven unique image conditions: *Elephant* or *Pizzas* mixed with 0%, 50%, or 75% noise, and a pooled 100% noise condition. First, each natural scene was converted to grayscale and transformed into the frequency domain using a 2D fast Fourier transform (FFT), producing a magnitude matrix and a phase matrix. The magnitude matrices from the two natural scenes were averaged to create a common magnitude matrix, ***M****.* Then to create images with different levels of noise, the phase matrix from either *Elephant* or *Pizzas* was mixed with a random noise matrix, whose values were drawn from a uniform distribution between 0 and 2*pi, at mixing ratios of 100:0 (original phase), 50:50, 25:75, or 0:100 (entirely noise). Each mixed phase matrix was combined with ***M***, and the final image was reconstructed using an inverse 2D FFT.

**Fig 2:**
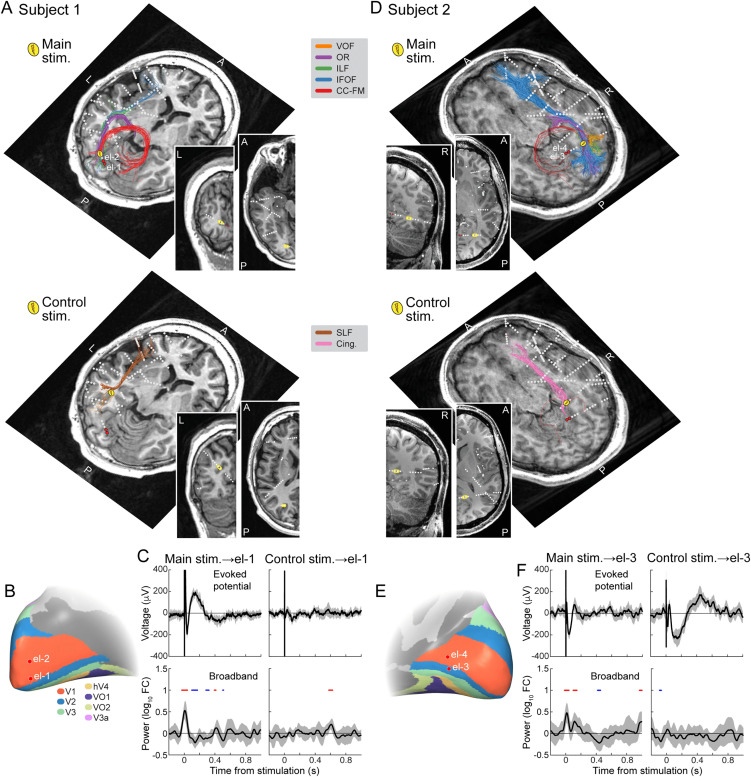
Stimulation sites and measurement electrodes in the EVC. **A,** Main and control stimulation sites, and measurement electrodes in the EVC (el-1 and el-2) in subject 1, plotted alongside white matter tracts within 4 mm of each stimulation site. VOF = vertical occipital fasciculus, OR = optic radiation, ILF = inferior longitudinal fasciculus, IFOF = inferior fronto-occipital fasciculus, CC-FM = corpus callosum forceps major, SLF = superior longitudinal fasciculus, Cing. = cingulum. Insets show coronal and axial T1-weighted MRI slices centered on each stimulation site. **B,** Measurement electrodes in the EVC on the subject’s inflated occipital pial surface. **C,** BSEPs (top) and stimulation-induced broadband changes (bottom) measured at el-1 from each stimulation site. Shaded intervals depict 95% confidence interval of the mean. Time points with mean broadband significantly greater than or less than 0 are highlighted in red and blue, respectively (one-sample *t*-test, P < 0.05). **D-F,** Stimulation sites and measurement electrodes in the EVC (el-3 and el-4) in subject 2, as in A-C. BSEPs and stimulation-induced broadband changes measured at el-2 and el-4 are shown in S1 Fig.

SPES (single biphasic pulses, 100 μs per phase, 6 mA) was delivered to target stimulation sites before the onset of each image (Figs 1, 2, S1). The electrical-visual time interval (EVI) between SPES and visual image onset was 200 ms, 100 ms, or 0 ms. Some trials included no stimulation (“sham” condition). The experiment was organized into 7-minute runs. Subject 1 performed 2 runs in one session and 2 additional runs 4 days later. Subject 2 performed 3 total runs in a single session. The 7 image conditions and 4 stimulation conditions (3 EVIs + sham) yielded 28 unique experimental conditions per stimulation site. There were 6–24 trials per experimental condition, yielding 216–384 total trials for each stimulation site.

The evoked potentials caused by image onset are referred to as visual evoked potentials (VEPs) and those caused by SPES are referred to as brain stimulation evoked potentials (BSEPs). By varying EVI in this task, we test how their combined evoked potential changes if visual inputs are modulated by SPES (Fig 1C, top). The analogous test is true for visual- and stimulation-induced broadband changes (Fig 1C, bottom).

### 2.4. Independent SPES data

To understand the brain’s responses to electrical stimulation alone, we first delivered SPES without visual stimuli or task instruction while each subject lay awake at rest, which yielded pure BSEPs and stimulation-induced broadband changes (Fig 2C, 2F). This experimental session occurred immediately after the first SPES+Visual session for subject 1 and immediately before the SPES+Visual session for subject 2. At each stimulation site, SPES was delivered 12 times (trials) with 2–5 second intervals between pulses, using the same stimulation parameters as in the SPES+Visual experiment. Since these data were collected under different experimental conditions, they were analyzed separately and not included in the main SPES+Visual analysis (Section 2.7).

### 2.5. Selection of measurement and stimulation electrodes

Electrode locations were identified from the postoperative CT scan and coregistered to the preoperative T1-weighted MRI [28], which was transformed into AC-PC space by affine transformations and trilinear voxel interpolation [29]. Measurement electrodes in the early visual cortex (EVC: V1, V2, V3) were anatomically identified (numbered 1–4, Fig 2) based on automated segmentation of subject T1-weighted MRIs using Freesurfer 7 [30], and the Benson and Wang probabilistic visual atlases [31,32]. Our primary analysis focused on these electrodes. Additional findings from visually responsive electrodes outside the EVC (identified functionally, see supplemental methods in S1 Text) are presented separately (in section 3.4 and S4-S5 Figs).

We stimulated electrode pairs located near major white matter tracts. The proximity of all electrodes to white matter tracts was estimated based on diffusion MRI (see supplemental methods in S1 Text). Whole brain tractography was performed using probabilistic tracking with constrained spherical deconvolution. From the whole brain connectome, fiber bundles were recognized using the pyAFQ [33] package with RecoBundles [34] and the 80 bundle HCP atlas [35]. We estimated how many fiber streamlines passed within 4 mm of each stimulation electrode and how many endpoints landed within 6 mm of each electrode in the EVC, for the following white matter tracts of interest: VOF = vertical occipital fasciculus, OR = optic radiation, ILF = inferior longitudinal fasciculus, IFOF = inferior fronto-occipital fasciculus, CC-FM = corpus callosum forceps major, SLF = superior longitudinal fasciculus, Cing. = cingulum. In each subject, the “main” stimulation site was located near a dense cluster of streamlines in the IFOF and ILF, which had endpoints in EVC. Stimulation sites located farther from the IFOF and ILF were selected as “control”.

### 2.6. iEEG data preprocessing

First, after visual inspection of all data, electrodes were excluded if they contained electrophysiological artifacts, were located outside of brain tissue, or were part of seizure onset zones per physician records. Individual trials containing epileptiform activity were also removed. Second, data were high-pass filtered (2^nd^ order Butterworth with cutoff frequency = 0.3 Hz, applied in a forward-reverse manner) to remove low-frequency drift. Third, data were re-referenced to an adjusted common average reference, calculated using the least responsive 25% of electrodes (based on activity 10–300 ms after visual onset) to minimize the introduction of large responses during re-referencing [36]. Finally, line noise at 60 Hz and its first two harmonics (120 and 180 Hz) were attenuated using a spectrum interpolation method [37], resulting in clean evoked potential data.

Time-varying time series of single-trial broadband responses (schematic in Fig 1A, green) were calculated from the clean evoked potential data as follows. Transient stimulation artifacts from SPES were removed by linear interpolation between 0 and 4 ms after stimulation. For each experimental condition (image × EVI), the mean evoked potential across trials was subtracted from each trial to minimize filtering artifacts due to sharp evoked potential peaks. For the independent SPES data, the mean evoked potential across all 12 trials was subtracted here. Each trial was forward-reverse filtered with third order Butterworth bandpass filters, divided into 20 Hz-wide bands between 70 and 170 Hz, excluding 110–130 Hz to avoid contamination by residual line noise harmonics. Power within each band was quantified as the squared envelope of the analytic signal (absolute value of the Hilbert transform); and the overall broadband power was the geometric mean across all bands. The broadband power was normalized by baseline, which was defined as the geometric mean broadband power across all sham stimulation trials 250–50 ms before visual onset, calculated separately for each run. For the independent SPES data, baseline was defined as 250–50 ms before stimulation. Final normalized units for broadband power were expressed as “fold increase” from baseline after subtracting 1. For example, this results in a 0.5-fold increase for a time point with broadband power = 9 µV^2^ and baseline = 6 µV^2^ (9÷6−1= 0.5). As the choice of iEEG reference may impact the estimation of broadband, we repeated broadband calculations and its associated analyses on bipolar re-referenced data, where the adjusted common average referencing step was replaced by the difference in evoked potentials between adjacent measurement electrodes (see supplemental results in S1 Text).

Time-frequency analysis was performed using continuous wavelet transformation of the single-trial evoked potential data. See details in supplemental methods (S1 Text).

### 2.7. Finite impulse response analysis of stimulation and visual responses

Since stimulation occurs shortly before visual onset in each trial, the stimulation and visual responses at least partially overlap in time. To separate the stimulation and visual component responses, we used finite impulse response (FIR) modeling, first for evoked potentials and then for broadband responses. These models test whether the observed responses are better explained by visual component responses that remain unchanged with stimulation (reflecting a pure additive relationship between stimulation and visual responses) or by visual component responses that change with varying EVI (reflecting an interaction between stimulation and visual responses). Simply put, for the same observed data, which independent predictors best explain the observed trial-to-trial differences? All modeling is performed on single-trial data.

**Fig 3:**
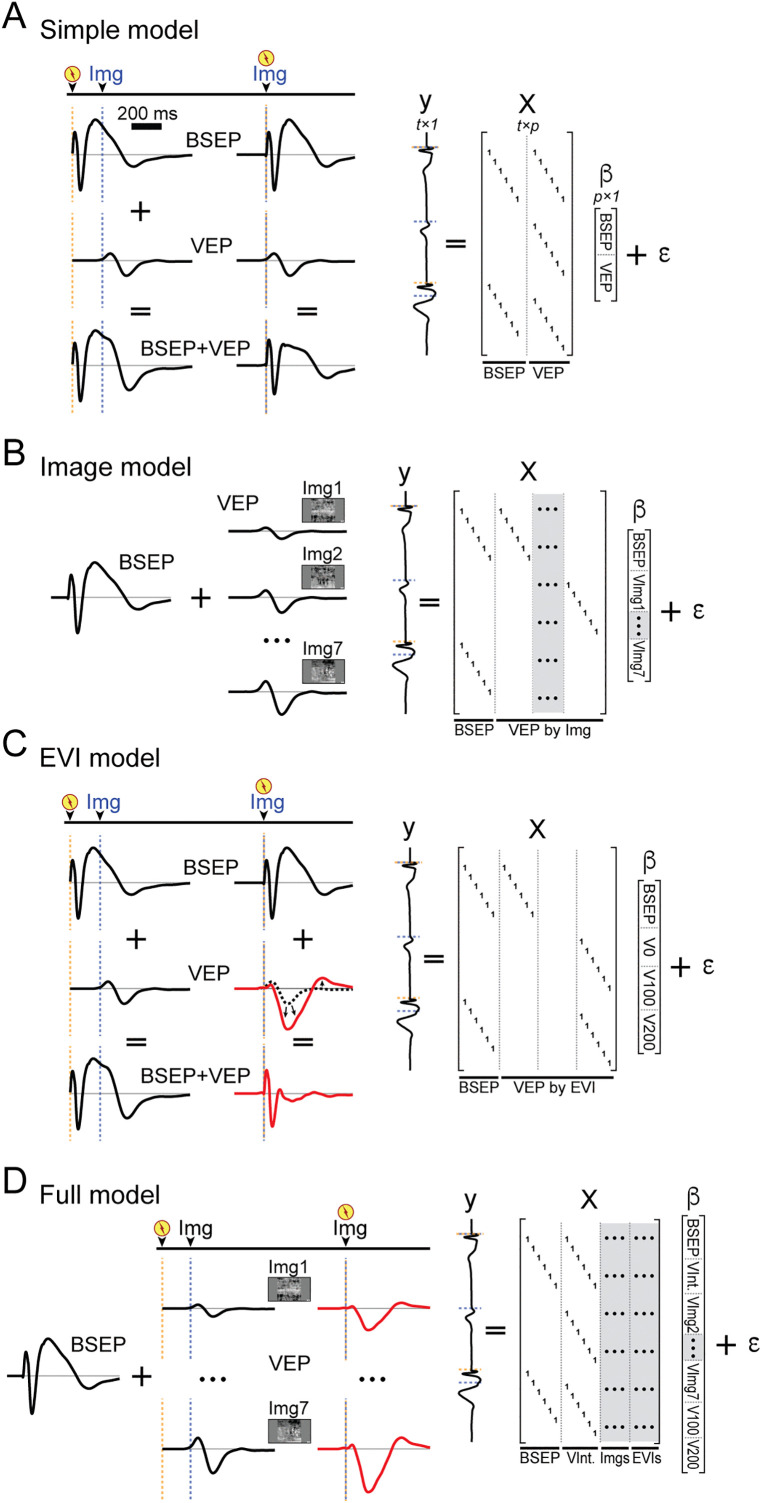
FIR models fit on SPES+Visual evoked potentials. **A,** The simple model fits a single set of BSEP predictors and a single set of VEP predictors, regardless of image condition or EVI. **B,** The image model fits independent VEP predictors for each of the seven image conditions. “...” denotes multiple condensed predictor sets. **C,** The EVI model fits independent VEP predictors for each of the three stimulation conditions (sham is merged with 200 ms). **D,** The full model fits nine independent sets of VEP predictors, corresponding to all image and stimulation conditions.

Here, we describe the FIR analysis for evoked potentials, which includes FIR modeling, model comparison, and final model selection steps. The FIR analysis for broadband responses followed a similar procedure with a few key differences (see supplemental methods in S1 Text and S6 Fig for details). First, single-trial evoked potential time series were downsampled to 600 Hz. We evaluated four FIR models, each expressed as a linear system:


$$\begin{array}{c} y=X\beta +\varepsilon , \end{array}$$

where *y* is the concatenated vector of all observed single-trial evoked potentials with dimensions *t ×* 1; *X* is the design matrix, with dimensions *t × p,* containing finite impulse response predictors (zeros and ones) for hypothesized component evoked potentials (columns, stimulation or visual) across time points; *β* is the unknown weights for voltage across time points for the concatenated component responses, with dimensions *p ×* 1, to be estimated via least squares regression (Fig 3); and ε is the per-time point error between predictions and observed data. Across subjects and stimulation sites, *t* ranged from 129,923–287,633 (751 downsampled time points *×* 173–383 trials); and across the four FIR models (see below), *p* ranged from 1200 to 6000 (600 predicted time points *×* 2–10 sets of predictors).

The four FIR models include different sets of predictors in the design matrix, *X*, and their corresponding estimates in *β*. The input data, *y*, do not vary across models. Here we subdivide *X* and *β* by predictor sets for each model, for clarity. The first and second FIR models assume that all observed evoked potentials are the sum of a BSEP and VEPs that do not change with stimulation:

1. For the simple model, *X* contains two sets of predictors: one set for the BSEP 0–1 s post-stimulation ($X_{BSEP}$), and one set for the VEP 0–1 s after visual onset ($X_{VEP}$), regardless of EVI or image identity (Fig 3A):

$$\begin{array}{c} y=X_{BSEP}\beta_{BSEP}+X_{VEP}\beta_{VEP}+\varepsilon . \end{array}$$

1. For the image model, *X* contains eight sets of predictors – one set for the BSEP ($X_{BSEP}$) and one set of VEP predictors for each image condition ($X_{VEP_{img\text{1}-\text{7}}}$) – which allow VEPs to vary by image (Fig 3B):

$$\begin{array}{cc} y=\; & X_{BSEP}\beta_{BSEP}+X_{VEP_{img\text{1}}}\beta_{VEP_{img\text{1}}}+X_{VEP_{img\text{2}}}\beta_{VEP_{img\text{2}}}+X_{VEP_{img\text{3}}}\beta_{VEP_{img\text{3}}}+X_{VEP_{img\text{4}}}\beta_{VEP_{img\text{4}}}\\ & \;\;+X_{VEP_{img\text{5}}}\beta_{VEP_{img\text{5}}}+X_{VEP_{img\text{6}}}\beta_{VEP_{img\text{6}}}+X_{VEP_{img\text{7}}}\beta_{VEP_{img\text{7}}}+\varepsilon . \end{array}$$

The third and fourth models allow visual responses to vary by stimulation condition:

1. For the EVI model, *X* contains four sets of predictors: one set for the BSEP ($X_{BSEP}$), and one set of VEP predictors for each EVI ($X_{VEP_{EVI\text{0}}}$, $X_{VEP_{EVI\text{1}\text{0}\text{0}}}$, $X_{VEP_{EVI\text{2}\text{0}\text{0}}}$) (Fig 3C). Because four stimulation conditions (sham and three EVIs) constrain the EVI model to a maximum of four independent sets of predictors (one BSEP and three EVI-dependent VEPs), sham trials were assigned the same VEP predictors as the 200 ms EVI condition ($X_{VEP_{EVI\text{2}\text{0}\text{0}}}$) to avoid an underdetermined system:

$$\begin{array}{c} y=X_{BSEP}\beta_{BSEP}+X_{VEP_{EVI\text{0}}}\beta_{VEP_{EVI\text{0}}}+X_{VEP_{EVI\text{1}\text{0}\text{0}}}\beta_{VEP_{EVI\text{1}\text{0}\text{0}}}+X_{VEP_{EVI\text{2}\text{0}\text{0}}}\beta_{VEP_{EVI\text{2}\text{0}\text{0}}}+\varepsilon . \end{array}$$

1. For the full model, *X* contains ten sets of predictors: one set for the BSEP ($X_{BSEP}$), and nine sets of VEP predictors in a two-way factorial design across image condition and EVI. The first set of VEP predictors serves as a two-way “intercept” term ($X_{VEP_{\text{0}}}$), which by itself represents the [0 ms EVI, 100% image noise] condition, and which is present on all experimental conditions. The next six sets ($X_{VEP_{img\text{2}-\text{7}}}$) encode deviations from the intercept to the other six image conditions, and the final two sets ($X_{VEP_{EVI\text{1}\text{0}\text{0}}},\;X_{VEP_{EVI\text{2}\text{0}\text{0}}}$) encode deviations from the intercept to the 100 ms and 200ms/sham EVI conditions.

$$\begin{array}{cc} y=\; & X_{BSEP}\beta_{BSEP}+X_{VEP_{\text{0}}}\beta_{VEP_{\text{0}}}+\;{\;X}_{VEP_{img\text{2}}}\beta_{VEP_{img\text{2}}}+X_{VEP_{img\text{3}}}\beta_{VEP_{img\text{3}}}+X_{VEP_{img\text{4}}}\beta_{VEP_{img\text{4}}}\\ & \;\;+X_{VEP_{img\text{5}}}\beta_{VEP_{img\text{5}}}+X_{VEP_{img\text{6}}}\beta_{VEP_{img\text{6}}}+X_{VEP_{img\text{7}}}\beta_{VEP_{img\text{7}}}+\;X_{VEP_{EVI\text{1}\text{0}\text{0}}}\beta_{VEP_{EVI\text{1}\text{0}\text{0}}}\\ & \;\;+X_{VEP_{EVI\text{2}\text{0}\text{0}}}\beta_{VEP_{EVI\text{2}\text{0}\text{0}}}+\varepsilon . \end{array}$$

The four FIR models were compared, separately for each stimulation–measurement electrode pair. To control for the different numbers of free parameters in the models, we used split-half validation to quantify model accuracy. Odd-numbered trials within each experimental condition were pooled as training data for each FIR model, and the even-numbered trials were held out for testing. Model performance was assessed using the coefficient of determination (COD):


$$\begin{array}{c} COD\;=\;\text{1}-\frac{\sum_{t_{\text{1}}}^{t_{\text{2}}}(V_{k}-f_{k}{)}^{\text{2}}}{\sum_{t_{\text{1}}}^{t_{\text{2}}}(V_{k}{)}^{\text{2}}}, \end{array}$$

where *V*_*k*_ is the observed voltage at time point *k*, *f*_*k*_ is the predicted voltage at time point *k*, *t*_*1*_ is the earliest predictor time point available based on stimulation condition (e.g., *t*_*1*_ = -0.2 s if EVI = 200 ms, *t*_*1*_ = 0 s if sham or EVI = 0 ms, relative to visual onset), and *t*_*2*_ is fixed at 0.5 s after visual onset, which approximated the end of most meaningful evoked potentials.

The models we designed are naturally ordered in terms of complexity, and we used split-half validated COD to determine whether any of the more complex models are necessary to explain the data. Whenever the simple model had the highest mean COD or no other model was significantly better, we concluded that the simple model best described the data. Whenever the higher-performing of the image or EVI models significantly outperformed the simple model (by paired *t*-test on COD, right-tailed P < 0.05) but was not significantly outperformed by the full model, we concluded that this model best described the data, indicating that EVI or image identity was a significant predictor of visual response. The image and EVI models were not directly compared to each other. Finally, whenever the full model significantly outperformed the next best model, we concluded that the full model best described the data, indicating that both EVI and image identity were significant predictors of visual response. This selective hierarchical comparison approach was employed to minimize the total type 1 error. For baseline reference, COD was also computed directly from the data itself, where the prediction for each testing trial was simply the mean across training trials of the same experimental condition.

We checked whether button presses influenced evoked potentials using additional FIR predictors, but did not find that such a model explained additional variance. We calculated bootstrapped confidence intervals for all FIR voltage coefficients (β). These steps are described in the supplemental methods in S1 Text.

### 2.8. Psychometric analysis

We recorded the reaction times and accuracies of button presses. Psychometric analysis to determine factors that influenced reaction time and accuracy are described in detail in supplemental methods and results (S1 Text).

## 3. Results

To understand how single pulse electrical stimulation (SPES) affects ongoing neural processing, we stimulated electrodes in the white matter projecting to early visual cortex in two human subjects. The degree of interaction between stimulation and visually driven iEEG responses in the early visual cortex was assessed in order to test whether stimulation modulates image processing. We assessed two iEEG signal features that provide complementary information about neuronal activity: evoked potentials and broadband power changes.

### 3.1. Stimulation of visual white matter pathways produces evoked potentials and broadband power changes at electrodes in the early visual cortex

We first stimulated visual and control pathways in the absence of any task to examine the neurophysiologic effects produced by stimulation alone. SPES was delivered at two stimulation sites in each subject: a main stimulation site near major visual pathways (<4 mm from the OR, VOF, ILF, IFOF, and CC-FM), and a control stimulation site located near the SLF and cingulum bundles. Measurement electrodes were located in V1-V2 (electrodes 1–2 in subject 1, Fig 2A-2B, electrodes 3–4 in subject 2, Fig 2D-2E). Diffusion MRI confirmed that streamlines connect measurement electrodes to the main stimulation site, but not to the control stimulation site. Specifically, of all streamlines near the main stimulation sites, 23 (17%), 17 (12%), 8 (1.3%), and 11 (1.8%) passed within 6 mm of measurement electrodes 1–4, respectively.

Brain stimulation evoked potentials (BSEPs) did not correlate perfectly with anatomical connectivity, as stimulation of the main sites in both subjects as well as the control site in subject 2 produced prominent BSEPs in EVC (Figs 2C and 2F, S1 top). In contrast, stimulation-induced broadband changes did correlate with anatomical connectivity, as they were prominent in EVC when stimulating the main sites but generally weak or absent when stimulating the control sites (Figs 2C and 2F, S1 bottom). These broadband changes were characterized by transient power increase within 50 ms of stimulation, followed by a return to baseline and then relative suppression lasting up to 400 ms in subject 1. Time-frequency analysis of the data confirmed these stimulation-induced broadband changes, and showed possible increases in low gamma range power (~20–50 Hz) when stimulating the main sites (S10 Fig). These broadband and spectral patterns resemble what we previously observed with SPES [18], and demonstrates that stimulation affects the local neuronal population activity selectively at connected sites.

### 3.2. SPES in visual pathways modulates visual evoked potentials

**Fig 4:**
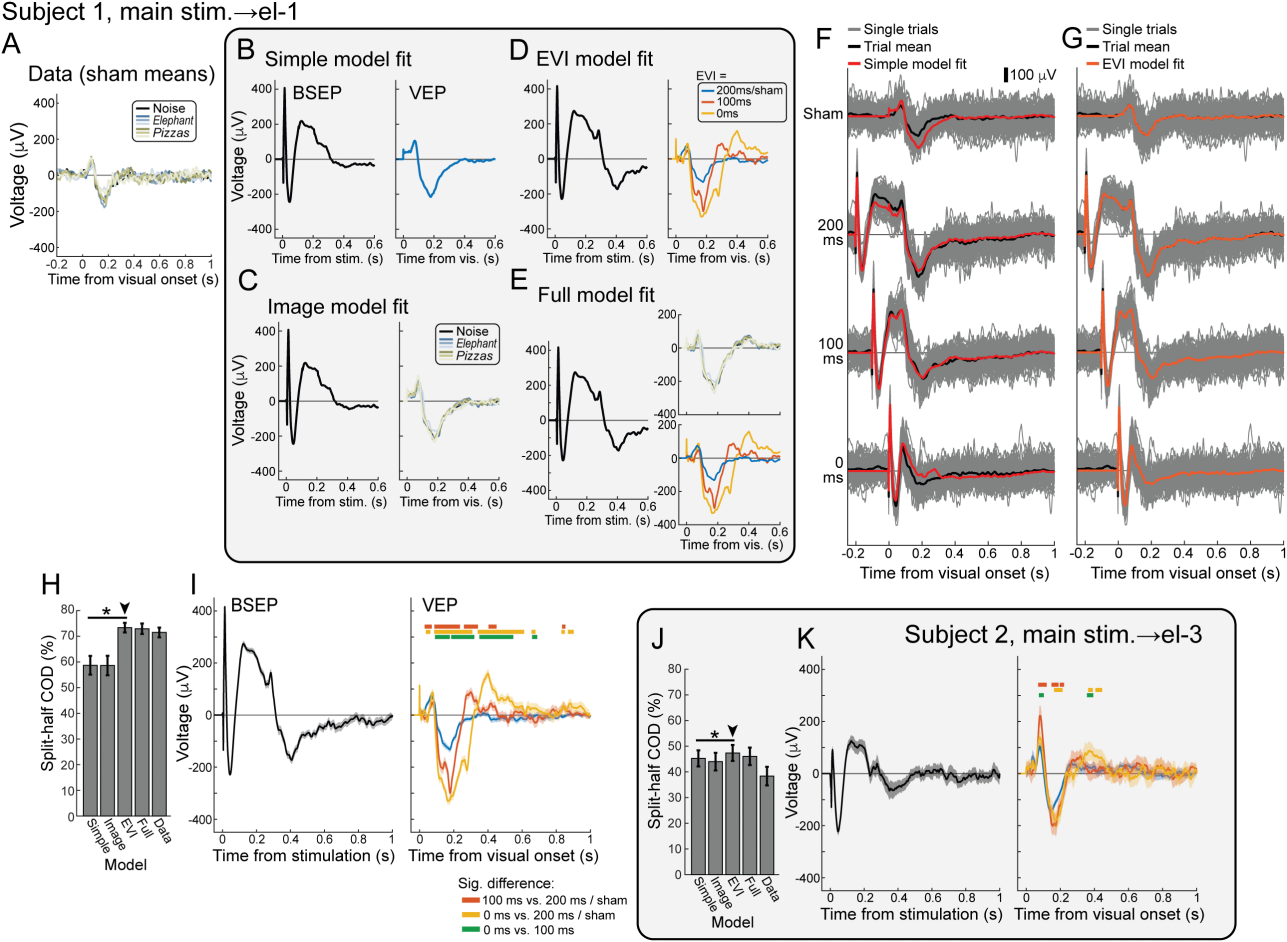
SPES in visual white matter tracts modulates VEPs at measurement electrodes in the EVC. **A,** Mean of VEPs across sham stimulation trials at el-1, for each image condition. **B-E,** Component responses from simple, EVI, image, and full models fit on evoked potential data at el-1 from the main stimulation site in visual white matter. In the image model legend, lighter color denotes lower image noise (75%, 50%, 0%). **F,** Comparison between the data and the predictions from the simple model (B) at each EVI. All image conditions are pooled for each row. **G,** Comparison between the data and predictions from the EVI model (D) at each EVI. **H,** COD (mean and standard error) from testing trials for models in B-E. “Data” predicts test trials using the mean of condition-matched training trials. The EVI model yielded the highest COD, significantly greater than the simple model (*paired *t*-test, right-tailed P < 0.05) **I**, Bootstrapped mean and 95% confidence intervals for EVI model responses (D). Significantly different time intervals between pairs of VEPs fit for each EVI are labeled with colored bars (Bootstrapped Differences, P < 0.01). **J, K,** COD and EVI model responses (mean and 95% confidence interval) for evoked potential data at analogous main stimulation site to measurement electrode el-3 in subject 2. COD and EVI model responses for el-2 and el-4, as well as for the control stimulation sites, are presented in S2 Fig.

The effects of single electrical pulses on visual processing were assessed by the SPES+Visual task, in which the white matter pathways were stimulated either immediately before or concurrently as subjects saw grayscale images with varying levels of noise (Fig 1A, 1B). SPES was delivered at electric-visual time intervals (EVI) of 200 ms, 100 ms, and 0 ms, except in interleaved sham stimulation trials. We used FIR models to separate brain stimulation (BSEP) and visual (VEP) evoked potential components from the overall observed responses (Fig 4B-4E). The BSEP components resembled the BSEPs recorded in the independent SPES task (Fig 2C), but with slightly greater amplitudes (direct comparison in S9 Fig). Separately, visual images elicited robust VEPs in early visual cortex when no stimulation was delivered (“sham”, e.g., subject 1: Fig 4A). Then, to determine whether these VEPs were modulated by SPES, we examine whether the VEPs differed significantly by EVI.

The models show that VEPs differed significantly by EVI when visual white matter pathways (main stimulation sites) were stimulated (subject 1: Fig 4D, 4E, 4I, subject 2: 4K), but not when the control sites were stimulated (S2B Fig). When visual pathways were stimulated, the EVI model (which allows the visual response to vary with EVI) explained a significantly greater fraction of variance in the observed data (subject 1: COD = 73.4%, subject 2: COD = 47.4%, Fig 4H, 4J) than the simple model. The evoked potentials predicted by the EVI model (Fig 4G) indeed align better with observed data than those predicted by the simple model (Fig 4F). In contrast, VEPs did not vary by image noise level, as neither the image model nor the full model outperformed the EVI model. In all cases, the best-fit model also explained more variance in the data than unmodeled predictions calculated from the means of condition-matched training trials (“data”) (Fig 4H, 4J). In contrast, suboptimal models sometimes performed worse than data (e.g., simple and image models in 4H). In summary, the models demonstrate that a single electrical pulse delivered at or before visual image onset can rapidly modulate VEPs in the EVC.

How exactly were the VEPs modulated? In both subjects, the prominent negative peak at ~180 ms after visual onset were larger when stimulation was delivered at or briefly before visual onset (EVI = 100 ms and EVI = 0 ms), compared to when stimulation was delivered 200 ms before visual onset or when there was no stimulation (Fig 4I, 4K). In subject 2, we note that stimulation at 100 ms before visual onset also significantly enhanced the amplitude of the initial positive VEP peak (at ~100 ms after visual onset, Fig 4K).

### 3.3. SPES in visual pathways suppresses broadband power without modulation of visual responses

Whereas evoked potentials reflect synchronous synaptic inputs, induced broadband power changes capture asynchronous changes in local neuronal input firing rates [19–21]. We tested whether SPES also modulates visual broadband responses by comparing the same four FIR models applied to the observed broadband power changes.

**Fig 5:**
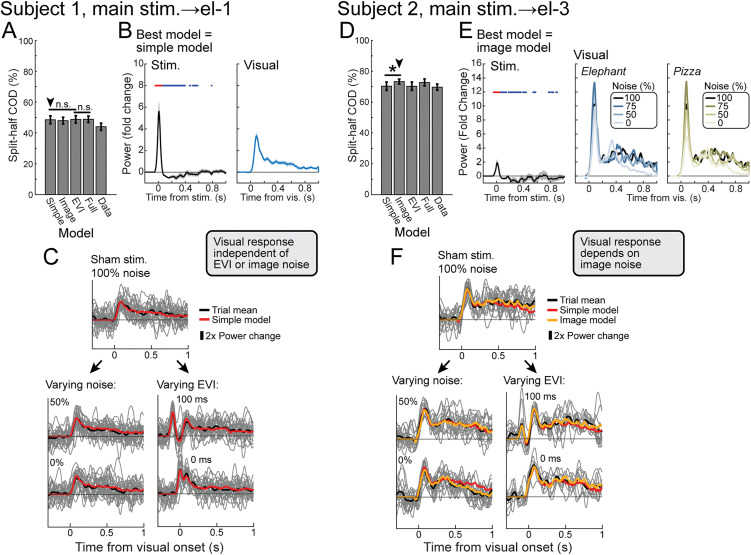
Independent stimulation-induced and visual induced broadband power changes at measurement electrodes in the EVC. **A,** COD (mean and standard error) from testing trials for each model fit on broadband power at el-1 from main site stimulation. “Data” predicts test trials using the mean of condition-matched training trials. “n.s.” = not significant by paired *t-*test, right-tailed P > 0.05 (Full > EVI, EVI > Simple). **B,** Bootstrapped mean and 95% confidence interval for the best fit (simple) model responses. Time points significantly greater than or less than 0 are highlighted in red and blue, respectively, for the stimulation response. **C,** Comparison between data and simple model predictions. Top: all trials with sham stimulation and 100% noise. Bottom: varying image noise level or EVI independently. **D,** COD from models fit on broadband power at el-3 from main site stimulation. The image model yielded the highest COD, significantly greater than the simple model (*paired *t*-test, right-tailed P < 0.05). **E,** Bootstrapped means and 95% confidence intervals for the best fit (image) model responses (only means shown for the visual responses). **F,** Comparison between data and simple, image model predictions. The simple model predictions provide a poorer fit to the data means than the image model predictions, when varying image noise. Model outputs for electrodes 2 and 4 and control stimulation sites are presented in S3 Fig.

SPES induced similar broadband responses during the visual task (captured by the FIR stimulation component) as SPES during rest. For both subjects, stimulation in visual pathways produced a transient increase in broadband power, followed by a decrease below baseline lasting ~0.4 s in the EVC (Fig 5B, 5E).

Despite a prominent stimulation-induced broadband response, SPES in visual pathways did not modulate visual broadband responses as it did VEPs: the EVI model did not improve COD over the simple model (Fig 5A, 5D). Broadband responses were best described by the simple or image models. In subject 1, the simple model explained 48.5% of variance and was not significantly improved by any other model (Fig 5A). Thus, the single visual broadband response predicted by the simple model aligns well with the observed broadband responses, regardless of image noise level and EVI (Fig 5C). In subject 2, the image model explained significantly more variance (COD = 73.3%) than the simple model (Fig 5D), which means that, in this case, predictions varying by image noise level better represented the observed data (Fig 5F). Specifically, increasing image noise was associated with longer sustained broadband elevation (Fig 5E). In general, SPES at the control stimulation sites did not modulate visual broadband responses either. The sole exception was one bipolar re-referencing case (S3B Fig, control stimulation to bipolar measurement electrodes 1–2), where the full model marginally outperformed the image model. The relevance of this isolated finding is uncertain. See S1 Text for more detail on bipolar re-referenced findings. Stimulating at control sites also produced smaller stimulation-induced broadband responses than stimulating in visual pathways (S3B Fig).

Time-frequency analysis of the data, in the form of wavelet spectrograms, largely recapitulated these broadband effects of electrical stimulation on visual processing (S10 Fig). Visual responses were characterized by a transient increase in broadband power that tapered over time, accompanied by a sustained decrease in alpha band (8–12 Hz) power. At the main stimulation sites, electrical stimulation produced a superimposed transient broadband increase followed by prolonged suppression, visible in the t-statistic heatmaps comparing each EVI condition to sham. This suppression was less consistent across control stimulation sites. Stimulation at the main site in subject 1 additionally produced increased power in the low gamma range (~20–50 Hz), consistent with the time-frequency findings from the independent SPES data.

### 3.4. Effects of SPES on visual processing outside the early visual cortex

Here, we examine the effects of SPES on visual processing outside of the EVC to gauge the anatomical selectivity of stimulation effects. We analyzed data from measurement electrodes outside the EVC that showed a significant broadband response to images in sham trials (subject 1: S4 Fig, electrodes 5–10; subject 2: S5 Fig, electrodes 11–13). These electrodes were located in LO1, the precentral sulcus and the inferior frontal gyrus.

SPES significantly modulated visual evoked potentials only at the two measured LO1 electrodes (S4C Fig, electrodes 5 and 6). Here, we observed a weaker but similar pattern of modulation as at measurement electrodes in the EVC: stimulation with EVI = 0 or 100 ms yielded slightly larger VEPs than stimulation with EVI = 200 ms or sham stimulation. However, the modulation at electrode 6 occurred in response to stimulation at the control site, rather than the main site. Overall, these weaker effects may reflect feedforward propagation from EVC. Stimulation did not affect the visual broadband responses at any electrode.

In general, for a given stimulation–measurement pair, SPES could have three alternative effects on either broadband or evoked potentials. First, it could produce no significant stimulation response (by bootstrapping, see supplemental methods in S1 Text). Second, it could produce a significant stimulation response but fail to modulate the visual response (i.e., simple or image model fit best). Third, it could produce a significant stimulation response *and* modulate the visual response (i.e., EVI or full model fit best). The two-way effects of SPES on evoked potentials vs. broadband, across all stimulation–measurement pairs, are presented in Table 1. This shows that SPES which modulated VEPs was also more likely to induce a stimulation broadband response (Table 1, third row, column 2 > column 1), whereas SPES which failed to modulate VEPs was more likely to induce no broadband response (Table 1, second row, column 1 > column 2). Thus, SPES modulation of VEPs appears to roughly overlap with its ability to induce a stimulation broadband response.

**Table 1:** Cross-modal modulatory relationship for all stimulation→measurement electrode pairs.

BroadbandEP		-SPES, -mod.	+SPES, -mod.	+SPES, + mod.
-SPES, -mod.		Control→el-11 Main→el-12 Control→el-12	Main→el-11 Control→el-13	
+SPES, -mod.		**Control→el-2** **Control→el-4** Main→el-7 Control→el-7 Main→el-8 Control→el-8 Main→el-9 Control→el-9 Main→el-10 Control→el-10	**Control→el-1** **Control→el-3** Control→el-5 Main→el-6 Main→el-13	
+SPES, + mod.		Control→el-6	***Main→el-1*** ***Main→el-3*** **Main→el-2** **Main→el-4** Main→el-5	

Rows indicate the relationship between BSEPs and VEPs, and columns indicate the relationship between stimulation and visual induced broadband changes. “-SPES, -mod.” = Stimulation did not produce a significant response and did not modulate visual response; “+SPES, -mod.” = stimulation produced a response but did not modulate the visual response; “+SPES, +mod.” = stimulation modulated the visual response (best-fit model was EVI or full). Measurement electrodes in the EVC are bolded, and main stimulation-measurement electrode pairs shown in Figs 4 and 5 are additionally in italics.

## 4. Discussion

In this study, we tested the modulatory potential of single pulse electrical stimulation (SPES) on visual evoked neuronal activity. Specifically, we quantified the effects of SPES in white matter tracts on evoked potentials and broadband changes in the visual system, both at rest and during a visual discrimination task. We found that visual white matter stimulation produced high amplitude evoked potentials in the early visual cortex, as well as transient broadband power increase followed by a longer decrease below baseline. During the visual discrimination task, SPES affected evoked potentials and broadband power in different ways: whereas it modulated the shape and amplitude of visual evoked potentials, it did not modulate visual broadband responses but rather induced broadband changes that directly added to them.

### 4.1 Single pulse electrical stimulation enhances visual evoked activity

Our results show that a single electrical pulse delivered in the visual pathways could enhance closely timed visual evoked potentials (VEPs). The timing of the single pulse was important, as EVIs of 0 and 100 ms had distinct modulatory effects on the VEP. This parallels paired-pulse transcranial magnetic stimulation and intracranial stimulation findings, where the effect of a conditioning stimulus on a test stimulus depends too on the interstimulus interval – facilitation when ≤50 ms but inhibition when ≥100 ms [38]. Similar mechanisms might underlie these paired-pulse findings as ours. Visual evoked potentials are thought to be driven by synchronous inputs from the thalamus and other cortical areas [24,25]. These results therefore suggest that visual white matter stimulation may induce a brief neuronal response in the visual cortex that facilitates those synchronous inputs.

### 4.2 Single pulse electrical stimulation affects total broadband activity

At measurement electrodes in the EVC, the stimulation-induced broadband response was characterized by a transient, several-fold increase in power followed by a sustained decrease. This pattern resembled our previous findings in the ventral temporal cortex when SPES was delivered to the hippocampus [18], and is also consistent with previously reported effects of LGN stimulation on V1 [17].

The sustained broadband decrease from stimulation implies a relative suppression in total asynchronous neuronal firing in the visual cortex after the initial ~100 ms of image onset. Note that unlike evoked potentials, broadband power has no meaningful polarity, and so its decrease from baseline does not mean the superposition of a distinct, *negative* signal. Rather, suppression of ongoing broadband activity is a more likely explanation [17]. Mechanistically, the sustained stimulation-induced suppression could arise from refractory inhibition due to presynaptic neurotransmitter depletion, afterhyperpolarization, or postsynaptic receptor desensitization after the initial transient excitation [39–41].

The stimulation-induced broadband response was simply additive to the visual broadband changes. A consequence of this addition is that differences in total broadband power are observed depending on the electrical-visual interval (EVI). When stimulation and visual onset are simultaneous, the two transient broadband peaks “stack” to yield greater total broadband power immediately after visual onset. But when stimulation and visual onset are offset by 200 ms, the visual broadband peak is counteracted by the suppressive phase of the stimulation broadband response, to yield lesser total broadband power immediately after visual onset. This difference might be meaningful if total population activity in the visual cortex influences downstream processing.

### 4.3. Visual evoked potential modulation explained by added noise

While single pulse electrical stimulation had different effects on evoked potentials and broadband responses, our results showed that modulation of VEPs and a significant stimulation-induced broadband response often occurred together. This general pattern emerged at the group level across all visually responsive measurement electrodes in and out of the EVC: SPES that modulated the VEP more often than not produced a broadband response (Table 1, third row, column 2 > column 1), while SPES that did not modulate the VEP more often than not did not produce a broadband response (Table 1, second row, column 1 > column 2). This suggests that a shared underlying mechanism, like added noise, may explain both effects.

If a single electrical pulse adds noise to a downstream neuronal population, our data may be explained by the phenomenon of stochastic resonance [12,15]. In stochastic resonance, a small amount of random noise can boost periodic or event-related inputs above a neuronal firing threshold. The net effect is an increase in the synchrony and amplitude of signals at the neuronal population level. Too much noise, however, would drown the signal. Since broadband power is thought to reflect Poisson-distributed, noisy neuronal inputs [20], the addition of more broadband power after visual onset by the stimulation-induced transient may be interpreted as the addition of random noise. And since evoked potentials are thought to reflect synchronous inputs, it follows that the VEPs after stimulation are amplified via stochastic resonance.

Added noise could be implemented at the pre- or post-synaptic level. At the presynaptic axon terminals of stimulated white matter tracts, SPES may cause a temporary buildup of Ca^2+^ that primes subsequent neurotransmitter release in response to visual stimuli [42–44]. Postsynaptically, additional excitatory NMDA receptors could be activated by glutamate spillover from the closely timed electrical and visual events [44,45]. Both potential mechanisms lead to larger excitatory postsynaptic potentials, which result in a larger VEP when summed across the neuronal population.

### 4.4. Alternative explanations for visual evoked potential modulation

Single pulse transcranial magnetic stimulation has been shown to reset the phase of ongoing low frequency oscillations in scalp EEG [46]. If SPES can achieve a similar result, it could align specific phases of ongoing oscillations with the subsequent visual inputs, potentially modulating neuronal responses to those inputs [47]. Currently, this is not well explored in SPES, and testing this potential explanation may require careful analysis of phase relationships after SPES and immediately before VEPs.

The observed VEP facilitation might also be explained by rebound hyperactivity in visual cortex neurons subsequent to a more rapid, transient suppression; Kara et al. [48] observed this phenomenon in cats when microstimulation was delivered to thalamic neurons with receptive fields close to measured V1 simple cells. However, an initial transient decrease in broadband power, not increase, would be expected immediately following stimulation. Thus, while rebound activity may be present in single cells, our population broadband measurements did not reveal such effects.

### 4.5. Single pulse electrical stimulation and behavioral threshold

Despite electrophysiologic evidence of visual facilitation, we observed inconsistent effects of stimulation on reaction time and no effect on response accuracy (see supplemental results in S1 Text, S7-S8 Figs, and S1-S2 Tables). This was expected and in line with other SPES experiments, since the charge delivered was titrated to be above the electrophysiologic response threshold [49], but below the threshold of consistent behavioral detection. Stimulation thresholds for modulating evoked potentials, broadband activity, and behavior likely differ from one another and require careful titration. Higher amplitude stimulation may improve behavioral detection but also activate a larger volume of tissue, potentially obscuring the desired neurophysiology. For example, low current delivered to monkey MT has been shown to bias motion detection towards the direction encoded by stimulated neurons, but higher current simply impaired overall performance [50]. Stimulating with pulse trains may also improve behavioral detection, like during clinical functional mapping of eloquent cortex [51]. However, different stimulation frequencies can have contrasting effects on neuronal firing rates, thus adding a parametric dimension to carefully consider [17,52]. Our goal is to first understand how the basic single pulse unit affects visual electrophysiology. Future research can then develop models linking these electrophysiological effects to behavioral outcomes from varying frequency and amplitude.

### 4.6. Limitations

The experiments in this study were conducted in two young male adult humans. This limits the generalizability of our findings, and a cautious interpretation is advised. The iEEG electrodes were placed clinically and not optimized to be identical in both subjects, though the evidence provided by imaging supports general anatomical similarity in key electrode locations between our two subjects (Fig 2).

Diffusion MRI showed that the main stimulation site in each subject richly overlapped the same set of white matter tracts with projections to the visual cortex (Fig 2A and 2D, top). One caveat is that the relative composition of individual tracts is different between the two subjects: for example, CC-FM was more strongly stimulated in subject 1 than subject 2, while IFOF was more strongly stimulated in subject 2 than subject 1. Because stimulation simultaneously activated several major white matter tracts, we cannot presently pinpoint which pathway(s) might be chiefly responsible for the modulatory effects. Polysynaptic pathways indirectly linking the stimulation and measurement sites may also be involved. Our primary measurement electrodes of interest (electrodes 1–4) were located in the early visual cortex (Fig 2B and 2E), based on segmentation with Freesurfer and the Benson probabilistic visual atlas. However, the visual stimulus captured by each electrode’s population receptive field likely differed since they were in different areas of the visual cortex. Nevertheless, stimulation had similar effects across the four electrodes and did not vary significantly by image. Of note, evoked potentials at electrodes 3 and 4 showed similar modulation (e.g., Figs 4J-4K vs. S2A), despite them being located in V2 and V1, respectively. Overall, this supports a stimulation robustness that is not strongly dependent on precise retinotopy.

Despite differences in electrode placement and white matter activated, we discovered similar evoked potential shapes, broadband responses, and time interval-dependent modulation in both subjects. We emphasize that our results were well validated within each subject. Our single-trial analysis (with 6–24 trials per experimental condition and 173–383 trials per electrode) provided robust confidence intervals for all estimates of model coefficients and goodness-of-fit, and thus each subject served as an independent test of the hypothesis. In addition to testing the generalizability of these findings on more subjects, future studies could provide greatest benefit by systematically testing a range of stimulation and measurement sites. This could elucidate the extent of modulation throughout the rest of the visual system and determine which white matter tracts might be most implicated. We recommend a similar multimodal approach as ours that leverages CT, structural MRI, and diffusion MRI coregistration to precisely identify where electrodes are in relation to structural and functional brain areas, and in relation to major white matter tracts.

Finally, as noted in the Methods, sham and 200 ms EVI trials were assigned the same VEP predictors to avoid an underdetermined FIR system. This may introduce some distortion in predicted component shapes, to the extent that the true sham and 200 ms VEPs differ. Nevertheless, this limitation is unlikely to undermine the primary evidence for VEP modulation, which rests on the significantly improved model goodness-of-fit when predictors were allowed to vary by stimulation condition. We recommend that future studies employ a greater diversity of EVIs to circumvent this limitation and more accurately constrain ground-truth component shapes.

## 5. Conclusion

The experimental combination of SPES and visual inputs allowed us to uniquely observe how a single electrical pulse affects the neuronal processing of independent information. SPES in white matter tracts affected the visual cortex in a way consistent with other studies, showing a brief increase in population neuronal activity followed by longer lasting inhibition. These stimulation-induced broadband changes directly added to visual induced broadband changes. In contrast, visual evoked potentials, representative of synchronous visual inputs, were facilitated by SPES. These two complementary aspects of iEEG provide evidence for a shared underlying mechanism, e.g., that electrical stimulation adds neuronal noise. Combining electrical and visual inputs like this may serve well to elucidate the basis for even more complex neurostimulation paradigms on neuronal processing.

## Supporting information

S1 Fig BSEPs and induced broadband changes at measurement electrodes 2 and 4. BSEPs (top) and induced broadband changes (bottom) recorded at el-2 in subject 1 and el-4 in subject 2 from main and control stimulation sites in each subject. Shaded intervals depict 95% confidence interval of the mean. Time points with mean broadband significantly greater than or less than 0 are highlighted in red and blue, respectively (one-sample *t*-test, P < 0.05).
(TIF)

S2 Fig Split-half COD and EVI model responses on evoked potential data for all other measurement electrodes in the EVC and control stimulation sites. **A,** Results for main stimulation sites to el-2 (subject 1) and el-4 (subject 2). **B,** Results for control stimulation sites to all measurement electrodes in the EVC. Statistical comparisons for COD indicate paired *t*-test at right-tailed α = 0.05 for the full model > EVI/image models, or for EVI/image models > the simple model. Arrowheads indicate the best model. Shaded intervals show 95% confidence intervals of the mean, and significantly different time intervals between pairs of EVI conditions are labeled with colored bars above (bootstrapped differences, P < 0.01).
(TIF)

S3 Fig Split-half COD and best-fit FIR model on broadband power for all other measurement electrodes in the EVC and control stimulation sites. **A,** Results for main stimulation sites to el-2, el-4, and bipolar re-referenced electrode pairs, COD calculated up to 1 s after visual onset. **B,** Results for control stimulation sites to measurement electrodes in the EVC and their bipolar re-referenced pairs, COD calculated up to 1 s after visual onset. Statistical comparisons for COD indicate paired *t*-test at right-tailed α = 0.05 for the full model > EVI/image models, or for EVI/image models > the simple model. Arrowheads indicate the best model. Shaded intervals show 95% confidence intervals of the mean for the best model (omitted for image models). Time points significantly greater than or less than 0 are highlighted in red and blue, respectively, for stimulation responses. Lighter colors in image model responses denote lower noise (75%, 50%, 0%). **C, D,** COD calculated up to 0.5 s after visual onset.
(TIF)

S4 Fig FIR model fits on evoked potential and broadband data at visually responsive electrodes outside the EVC in subject 1. **A,** Visually responsive measurement electrodes outside the EVC in subject 1 (el-5–el-10) visualized on the subject’s inflated pial surface (top) and on subject coronal and axial T1-weighted MRI slices in red (bottom). Other electrodes within 4 mm of each slice are also plotted in white. **B,** Mean power spectral density plots for each visually responsive electrode across sham trials, before (rest) and after (image) visual onset. *R*^*2*^ is the variance of broadband log power explained by condition, image or rest. **C,** COD and best model responses for evoked potentials at measurement electrodes outside the EVC. s = simple, i = image, e = EVI, f = full, d = data. **D,** COD (calculated up to 1 s after visual onset) and best model responses for broadband power at measurement electrodes outside the EVC. Statistical comparisons indicate paired *t*-test at right-tailed α = 0.05 for the full model > EVI/image models, or for EVI/image models > the simple model. Arrowheads indicate the best model.
(TIF)

S5 Fig FIR model fits on evoked potential and broadband data at visually responsive electrodes outside the EVC in subject 2. **A,** Visually responsive measurement electrodes outside the EVC in subject 2 (el-11–el-13) visualized on the subject’s inflated pial surface (left) and on subject coronal and axial T1-weighted MRI slices in red (right). Other electrodes within 4 mm of each slice are also plotted in white. **B,** Mean power spectral density plots for each visually responsive electrode across sham trials, before (rest) and after (image) visual onset. *R*^*2*^ is the variance of broadband log power explained by condition, image or rest. **C,** COD and best model responses for evoked potentials at measurement electrodes outside the EVC. s = simple, i = image, e = EVI, f = full, d = data. **D,** COD (calculated up to 1 s after visual onset) and best model responses for broadband power at measurement electrodes outside the EVC. Statistical comparisons indicate paired *t*-test at right-tailed α = 0.05 for the full model > EVI/image models, or for EVI/image models > the simple model. Arrowheads indicate the best model.
(TIF)

S6 Fig FIR models fit better on broadband power than on broadband log power. **A,** An FIR model fit on log power is mathematically equivalent to a multiplicative model fit on power. **B,** (Left) image model responses fit on broadband power vs. broadband log power, for an example stimulation-measurement electrode pair and one image condition. (Right) Comparison between data and the predictions from power and log power models. **C,** (Top) Difference in split-half mean absolute error (MAE) between log power and power image models for each stimulation-measurement electrode pair. Gray dots are single experimental conditions, and red circle is mean across experimental conditions. (Bottom) Distribution of differences in split-half MAE, pooled across all stimulation-measurement electrode pairs. Mean MAE is greater for the log power model fits compared to the power model fits.
(TIF)

S7 Fig Reaction time and response accuracy improve with less image noise. **A,** Subject 1 response time for all trials, by image condition. **B,** Subject 1 response accuracy for each image condition. The number above each bar indicates the number of hits for that image condition. Chance level (50%) is shown with the gray dashed line. **C,** Response accuracy as a Weibull function of image noise level for each stimulation site. **D-F** show analogous results in subject 2 as in A-C.
(TIF)

S8 Fig Reaction times subdivided by image and stimulation condition. Expansion of distributions in S7A, S7D Figs, by stimulation condition. Each point is a single trial.
(TIF)

S9 Fig Comparison of BSEPs from independent SPES task and from SPES+Visual task. For each stimulation–measurement pair: observed BSEPs from the independent SPES task are shown in black (mean and 95% confidence interval), observed trials from the SPES+Visual task with EVI = 200 ms are shown in blue (mean and 95% confidence interval), and the stimulation evoked potential component predicted by the EVI model (from the SPES+Visual data) is shown in purple. The observed data in blue contain only stimulation effect on 0–200 ms, so it can be meaningfully compared to the other traces on this interval.
(TIF)

S10 Fig Time-frequency analysis of SPES and visual responses. For each stimulation–measurement pair: (Left) Average wavelet spectrogram for each stimulation condition of the SPES+Visual task, time-locked to visual onset. (Top right) Average wavelet spectrogram for the independent SPES task. For comparison, the broadband response from Fig 2C, 2F is copied above. (Bottom right) t-statistic heatmaps comparing each SPES+Visual EVI to sham, time-locked to stimulation. These heatmaps estimate the time-frequency effect attributable to SPES in the SPES+Visual task (i.e., stimulation component). For comparison, the broadband FIR stimulation component from Figs 5B, 5E, or S3B is copied above.
(TIF)

S1 Table Multivariate linear regression of reaction time. Multivariate linear regression of response time for each subject. The intercept term represents the expected response time in sham stimulation trials when the run number is 1, the trial onset is at 0 s, and the image is 100% noise.
(DOCX)

S2 Table Multivariate logistic regression of response accuracy. Multivariate logistic regression of response accuracy for each subject. The intercept term represents the expected response accuracy in sham stimulation trials when the run number is 1, the trial onset is at 0 s, and the image coherence is 0% (100% noise).
(DOCX)

S1 Text Supplemental methods and results. (DOCX)
