# Supplementary material for: Single pulse electrical stimulation in white matter modulates iEEG visual responses in human early visual cortex
Source: PLoS Comput Biol. 2026 Jul 24;22(7):e1014563. doi: 10.1371/journal.pcbi.1014563 (PMC13426923; doi:10.1371/journal.pcbi.1014563)
Supplement: S1 Table — Multivariate linear regression of response time for each subject. The intercept term represents the expected response time in sham stimulation trials when the run number is 1, the trial onset is at 0 s, and the image is 100% noise. (DOCX) [file pcbi.1014563.s011.docx]

**S1 Table. Multivariate linear regression of reaction time**

|  | **Coefficient** | **95% CI** | **Test Statistic** | **P-value** |
| --- | --- | --- | --- | --- |
| **Subject 1** | | | | |
| Intercept | 0.71 | [0.65, 0.76] | *t*(633) = 24.3 | 7.2e-93* |
| Run number | -0.018 | [-0.039, 0.0027] | *t*(633) = -1.71 | 0.088 |
| Trial onset time (s) | -2.1e-4 | [-3.3e-4, -8.3e-5] | *t*(633) = -3.29 | 0.0011* |
| Main-200ms | 0.018 | [-0.035, 0.070] | *t*(633) = 0.669 | 0.50 |
| Main-100ms | 0.045 | [-0.0072, 0.098] | *t*(633) = 1.69 | 0.091 |
| Main-0ms | -0.0064 | [-0.059, 0.046] | *t*(633) = -0.238 | 0.81 |
| Control-200ms | 0.013 | [-0.040, 0.065] | *t*(633) = 0.476 | 0.64 |
| Control-100ms | 0.018 | [-0.034, 0.070] | *t*(633) = 0.669 | 0.50 |
| Control-0ms | 0.0089 | [-0.043, 0.061] | *t*(633) = 0.337 | 0.74 |
| *Elephant*-75% noise | 0.015 | [-0.030, 0.060] | *t*(633) = 0.655 | 0.51 |
| *Elephant*-50% noise | -0.053 | [-0.097, -0.0077] | *t*(633) = -2.30 | 0.022* |
| *Elephant*-0% noise | -0.090 | [-0.13, -0.045] | *t*(633) = -3.92 | 9.7e-5* |
| *Pizzas*-75% noise | -0.0039 | [-0.049, 0.041] | *t*(633) = -0.172 | 0.86 |
| *Pizzas*-50% noise | -0.063 | [-0.11, -0.018] | *t*(633) = -2.77 | 0.0057* |
| *Pizzas*-0% noise | -0.11 | [-0.16, -0.069] | *t*(633) = -5.02 | 6.6e-7* |
| **Subject 2** | | | | |
| Intercept | 1.0 | [0.95, 1.1] | *t*(411) = 22.7 | 1.1e-74* |
| Run number | -0.12 | [-0.17, -0.080] | *t*(411) = -5.54 | 5.3e-8* |
| Trial onset time (s) | 1.9e-4 | [-2.0e-5, 4.0e-4] | *t*(411) = 1.78 | 0.076 |
| Main-200ms | 0.065 | [-0.038, 0.17] | *t*(411) = 1.24 | 0.22 |
| Main-100ms | 0.044 | [-0.057, 0.15] | *t*(411) = 0.861 | 0.39 |
| Main-0ms | 0.11 | [0.0079, 0.21] | *t*(411) = 2.12 | 0.035* |
| Control-200ms | -0.065 | [-0.14, 0.014] | *t*(411) = -1.62 | 0.11 |
| Control-100ms | -0.088 | [-0.17, -0.0090] | *t*(411) = -2.19 | 0.029* |
| Control-0ms | -0.048 | [-0.13, 0.030] | *t*(411) = -1.21 | 0.23 |
| *Elephant*-75% noise | -0.026 | [-0.10, 0.051] | *t*(411) = -0.671 | 0.50 |
| *Elephant*-50% noise | -0.25 | [-0.32, -0.17] | *t*(411) = -6.58 | 1.4e-10* |
| *Elephant*-0% noise | -0.29 | [-0.36, -0.21] | *t*(411) = -7.39 | 8.1e-13* |
| *Pizzas*-75% noise | 0.016 | [-0.062, 0.095] | *t*(411) = 0.407 | 0.68 |
| *Pizzas*-50% noise | -0.17 | [-0.25, -0.094] | *t*(411) = -4.41 | 1.3e-5* |
| *Pizzas*-0% noise | -0.30 | [-0.37, -0.22] | *t*(411) = -7.64 | 1.6e-13* |

Multivariate linear regression of response time for each subject. The intercept term represents the expected response time in sham stimulation trials when the run number is 1, the trial onset is at 0 s, and the image is 100% noise.
