# Supplementary material for: Single pulse electrical stimulation in white matter modulates iEEG visual responses in human early visual cortex
Source: PLoS Comput Biol. 2026 Jul 24;22(7):e1014563. doi: 10.1371/journal.pcbi.1014563 (PMC13426923; doi:10.1371/journal.pcbi.1014563)
Supplement: S2 Table — Multivariate logistic regression of response accuracy for each subject. The intercept term represents the expected response accuracy in sham stimulation trials when the run number is 1, the trial onset is at 0 s, and the image coherence is 0% (100% noise). (DOCX) [file pcbi.1014563.s012.docx]

**S2 Table. Multivariate logistic regression of response accuracy**

|  | **Ln OR** | **95% CI** | **Test Statistic** | **P-value** |
| --- | --- | --- | --- | --- |
| **Subject 1** | | | | |
| Intercept | -0.55 | [-1.3, 0.25] | *z* = -1.34 | 0.18 |
| Run number | 0.19 | [-0.11, 0.49] | *z* = 1.22 | 0.22 |
| Trial onset time (s) | 3.2e-5 | [-0.0018, 0.0018] | *z* = 0.034 | 0.97 |
| Scene: *Elephant* vs. *Pizzas* | -0.19 | [-0.57, 0.19] | *z* = -0.96 | 0.34 |
| Image coherence (%) | 0.033 | [0.026, 0.040] | *z* = 9.21 | 3.4e-20* |
| Main-200ms | 0.68 | [-0.10, 1.5] | *z* = 1.71 | 0.087 |
| Main-100ms | 0.53 | [-0.24, 1.3] | *z* = 1.35 | 0.18 |
| Main-0ms | -0.096 | [-0.84, 0.65] | *z* = -0.250 | 0.80 |
| Control-200ms | 0.13 | [-0.64, 0.89] | *z* = 0.320 | 0.75 |
| Control-100ms | -0.18 | [-0.93, 0.58] | *z* = -0.454 | 0.65 |
| Control-0ms | 0.10 | [-0.66, 0.86] | *z* = 0.257 | 0.80 |
| **Subject 2** | | | | |
| Intercept | -0.13 | [-1.1, 0.87] | *z* = -0.247 | 0.80 |
| Run number | -0.068 | [-0.58, 0.45] | *z* = -0.258 | 0.80 |
| Trial onset time (s) | 8.3e-4 | [-0.0016, 0.0033] | *z* = 0.667 | 0.50 |
| Scene: *Elephant* vs. *Pizzas* | -0.15 | [-0.65, 0.35] | *z* = -0.59 | 0.55 |
| Image coherence (%) | 0.039 | [0.029, 0.049] | *z* = 7.55 | 4.3e-14* |
| Main-200ms | -0.045 | [-1.2, 1.1] | *z* = -0.0739 | 0.94 |
| Main-100ms | -0.29 | [-1.5, 0.87] | *z* = -0.493 | 0.62 |
| Main-0ms | -0.30 | [-1.5, 0.85] | *z* = -0.514 | 0.61 |
| Control-200ms | -0.46 | [-1.4, 0.46] | *z* = -0.978 | 0.33 |
| Control-100ms | 0.21 | [-0.75, 1.2] | *z* = 0.430 | 0.67 |
| Control-0ms | -0.50 | [-1.4, 0.40] | *z* = -1.08 | 0.28 |

Multivariate logistic regression of response accuracy for each subject. The intercept term represents the expected response accuracy in sham stimulation trials when the run number is 1, the trial onset is at 0 s, and the image coherence is 0% (100% noise).
