# Supplementary material for: Single pulse electrical stimulation in white matter modulates iEEG visual responses in human early visual cortex
Source: PLoS Comput Biol. 2026 Jul 24;22(7):e1014563. doi: 10.1371/journal.pcbi.1014563 (PMC13426923; doi:10.1371/journal.pcbi.1014563)
Supplement: S1 Text — (DOCX) [file pcbi.1014563.s013.docx]

### Supplemental methods

#### Identification of visually responsive electrodes outside of early visual areas

Visually responsive measurement electrodes outside of early visual areas were functionally identified by their increased broadband power following visual onset during sham stimulation trials. Specifically, the power spectral density (PSD) was estimated using Welch’s method for the 500 ms intervals before and after visual onset, for all sham trials at each electrode. A mean log broadband power was calculated for each interval by averaging the log power of 1-Hz frequency bins from 70-170 Hz, excluding the 116-124 Hz bins to avoid contamination from the 120 Hz line noise harmonic. For each electrode, an *R^2^* value was computed as the fraction of variance explained by the binary categorical variable, before vs. after visual onset, across all intervals. Electrodes were classified as visually responsive if they showed higher average log broadband power after visual onset and a statistically significant *R^2^* > 0.1 (FDR-corrected F-test, P < 0.05).

#### Diffusion MRI acquisition and preprocessing

Diffusion MRI (dMRI) scan was performed in both subjects on a 3T Prisma MRI scanner (Siemens Healthineers, Forchheim, Germany). A series with two volumes at b = 0-100 s/mm^2^ and 60 directions at b = 1000 s/mm^2^ were acquired with TR = 5100 ms; TE = 71 ms; 46 slices at 4 mm thickness (zero gap), field of view of 220 mm and acquisition matrix of 128 x 128.

The T1-weighted and dMRI images were preprocessed using QSIprep. The T1-weighted image was corrected for intensity non-uniformity (1) and then skull stripped using ANTS 2.3.1. Spatial normalization to the ICBM 152 Nonlinear Asymmetrical template version 2009c (2) was performed through nonlinear registration with antsRegistration (3), after brain-extraction of both the T1-weighted image volume and the template. Brain tissue segmentation was performed using FAST (FSL 6.0.3:b862cdd5 (4)).

Any dMRI images with a b-value less than 100 s/mm^2^ were treated as a b = 0 image. MP-PCA denoising was applied with a 5-voxel window (5), and B1 field inhomogeneity was corrected using dwibiascorrect (MRtrix3) with the N4 algorithm (1). The mean intensity of the dMRI series was adjusted to match across b = 0 images from different dMRI scans. Head motion and eddy current were corrected using FSL’s eddy (version 6.0.3:b862cdd5, q-space smoothing factor = 10, 5 iterations, 1000 voxels used to estimate hyperparameters (6)). Linear first and second level models were used to characterize eddy current-related spatial distortion and eddy’s outlier replacement was run (7). For outlier detection, data were grouped by slice, only including slices with at least 250 intracerebral voxels. Groups deviating by more than 4 standard deviations from the prediction value were replaced with imputed values. Final interpolation was performed using the jac method. The dMRI time-series were then resampled with 1.25 mm isotropic voxels in AC-PC space.

#### Confidence intervals for finite impulse response coefficients

Each FIR model was bootstrapped (1000 times for evoked potentials and 200 times for broadband responses) to calculate per-time point confidence intervals for stimulation and visual component coefficients. Resampling was done within each experimental condition to maintain balanced trial counts. In the EVI model, bootstrapped differences between pairs of visual responses were analyzed to locate time intervals showing significant EVI-dependent modulation: Consecutive time points spanning ≥20 ms were considered significantly different between pairs of EVI conditions (e.g., 0 ms vs. 100 ms) if the 99% confidence interval for the differences excluded 0 at all time points. 20 ms was chosen because it approximates the duration of meaningful evoked potential features, such as the “N1” component in BSEPs (8). For the analysis in section 3.4, a stimulation response was considered significant only if ≥20% of total time points within the first 500 ms post-stimulation had 95% confidence intervals that excluded 0. These criteria assist in distinguishing clearly prominent and long-lasting stimulation responses from weaker/transient ones.

#### Finite impulse response analysis of broadband responses

FIR analysis on broadband-transformed SPES+Visual data proceeded similarly as that on evoked potential data. Broadband power was downsampled to 200 Hz and temporally smoothed in log space with 50 ms time windows before analysis.

A key decision in broadband analysis is whether to fit models on power or log power, as addition in log power corresponds to multiplication in power space (S6A Fig), and both approaches have precedence in the literature (9,10). Fitting on log power is statistically convenient because log power is approximately normally distributed whereas (raw) power is right-skewed; however, independent signals are theoretically additive in power when their phases are random (10). To guide this decision, we compared the mean errors of image models fit on power vs. log power across all stimulation–measurement electrode pairs. Models estimated responses using least absolute deviation (LAD) regression, rather than least squares regression, because LAD regression targets the trial median – a quantity equivalent between power and log power up to the transformation itself. Trials were split into training (odd) and testing (even) for each experimental condition, and the training trials were used to fit image models on power and on log power (S6B Fig). We then calculated the mean absolute error (MAE) of power and log power model predictions, relative to the median observed testing trial for each experimental condition. We compared MAE between power and log power models across all 28 experimental conditions for all stimulation–measurement electrode pairs. On average, fitting on power yielded significantly lower MAE than fitting on log power (S6C Fig, paired *t*-test, P < 0.05). Thus, we moved forward using power for the broadband FIR analysis.

The same FIR models applied to evoked potentials were applied to the observed broadband power changes, with a few minor differences. Each model (simple, image, EVI, full) was again a linear system:

$y=X\beta+\varepsilon$,

where *y* is the concatenated vector of all observed single trial broadband changes with dimensions *t ×* 1; *X* is the design matrix, with dimensions *t × p,* containing finite impulse response predictors for hypothesized component broadband responses (stimulation or visual); *β* is the unknown weights for power across time points for the concatenated component responses to be estimated, with dimensions *p ×* 1; and $\varepsilon$ is the per-time point error between predictions and observed data. Instead of least squares regression used for evoked potentials, we continued to used LAD regression to solve for *β*, as it is more robust to the right-skewed distribution of broadband power. Each set of predictors in *X* begins at 50 ms before stimulation or visual onset to account for temporal smoothing and smearing caused by forward-backward filtering in broadband preprocessing. The dimensionality was lower than for evoked potential data due to greater downsampling: *t* ranged from 52200 to 99963 (261 time points *×* 200–383 trials) and *p* ranged from 420 to 2100 (210 predicted time points *×* 2–10 sets of predictors).

To maintain consistency with the evoked potential analysis, we again calculated COD on testing trials to compare the model performances. Before COD calculation, broadband predictions and observations were log-transformed to approximate normality and ensure the robustness of COD. As broadband changes appeared to persist longer than evoked potentials, we extended the time window of COD calculation to 1 s after visual onset. COD calculated up to 0.5 s, consistent with evoked potential results, are additionally presented in S3C-S3D Fig.

#### Evaluating effects of button press in finite impulse response models

To account for possible premotor or motor activity associated with button press, we added 2 additional sets of predictors to each best-fit FIR model, time-locked to button presses for each hand. These predictors were fit from -0.2 to 0.5 s around button press for evoked potentials and -0.25 to 0.5 s for broadband responses. In subject 1, these predictors also accounted for possible brain activity associated with the auditory feedback synchronized with button press. A significant increase in COD would indicate evoked potentials or broadband changes attributable to button press activity, but neither was observed.

#### Time-frequency analysis

Spectrograms were computed by continuous wavelet transformation of the independent SPES and SPES+Visual evoked potential data, to complement the broadband analysis and to explore time-frequency effects of electrical and visual stimulation. Stimulation artifacts and mean evoked potentials (per experimental condition) were first removed from each trial of evoked potential data as described for broadband preprocessing in Section 2.6 of the main text. Single-trial spectrograms were then computed using the MATLAB R2023a cwt function, with an analytic Morlet wavelet (ω₀ = 6, 10 voices per octave) over a frequency range of 1.35-200 Hz, with power at each time-frequency bin defined as the squared amplitude of the wavelet coefficients. Power was normalized to baseline (geometric mean on 250-50 ms before visual onset, per frequency per run, similar to broadband normalization). Average spectrograms for each stimulation condition were calculated by taking the geometric mean across trials per time-frequency bin. To isolate the effects of electrical stimulation on SPES+Visual at each EVI condition (0, 100, 200 ms), we first subtracted from each stimulation trial the expected log power of sham trials with matching image condition. These per-trial log power differences were then tested against zero by one-sample t-test at each time-frequency bin, and the resulting heatmap of t-statistics quantifies the direction and significance of stimulation-related power differences across the time-frequency plain.

#### Psychometric analysis

We recorded reaction times and accuracies for each subject. Trials without responses were omitted. To examine whether reaction times differed across the seven image conditions, we performed a one-way ANOVA. Additionally, a multivariate fixed effects linear regression model was created to evaluate whether reaction time varied with run number, trial onset time, image condition (categorical), and stimulation condition (categorical). Stimulation condition had seven levels: sham, and stimulation at either main or control stimulation site at each EVI (0, 100, 200 ms).

For each image condition, a right-tailed binomial test determined whether response accuracy was significantly higher than expected by chance (50%). For each stimulation condition, we modeled response accuracy as a function of image noise using the best-fit Weibull function:

$Y =0.5 + 0.5(1-exp(-(x/\lambda)^{k})$,

where *Y* is the response accuracy between 0 and 1, *x* is the image coherence (the complement of image noise, 100% − image noise), and 𝜆 and *k* are parameters. For this analysis, trials were pooled across *Elephant* and *Pizzas* images of the same image noise level, resulting in four distinct levels in the independent variable (100%, 75%, 50%, or 0% image noise). For levels of 75%, 50%, and 0% image noise, we explicitly tested whether response accuracy depended on stimulation condition using Chi-Square tests of independence. We also fit a multivariate logistic regression model to test whether response accuracy varied with run number, trial onset time, the image scene contained (*Elephant* or *Pizzas*), image coherence, and stimulation condition.

### Supplemental results

#### SPES did not consistently impact reaction time or response accuracy

The main goal of the experiment was to test how a single electrical pulse modulates evoked and induced visual neuronal activity. However, it is valuable to assess whether a single electrical pulse influences perceptual measurements.

Subjects were asked to press a button to indicate whether noise masked images showed an *Elephant* or *Pizzas.* Mean reaction time differed significantly across the seven image conditions (subject 1: one-way ANOVA, *F*(6, 641) = 7.71, P = 5.1*10^-8^; subject 2: one-way ANOVA, *F*(6, 419) = 20.2, P = 8.4*10^-21^). As expected, the mean reaction time was significantly shorter for images with less noise (S7A, S7D Fig). To test whether reaction time was affected by stimulation, we fit a multivariate linear regression model, which adjusted for the run number, the trial order/timing, and the image category (S1 Table, S8 Fig). In subject 2, stimulation at the main site simultaneous with visual onset increased reaction time by 110 ms on average (*t*(411) = 2.12, P = 0.035), while stimulation at the control site 100 ms before visual onset decreased reaction time 88 ms on average (*t*(411) = -2.19, P = 0.029). However, these effects were not found in subject 1. Both subjects showed significant learning effects: subject 1 responded faster across trials within each run (*t*(633) = -3.29, P = 0.0011), and subject 2 responded faster with subsequent runs (*t*(411) = -5.54, P = 5.3*10^-8^).

Response accuracy increased with less image noise, and it was significantly above chance level for 75%, 50%, and 0% noise images (S7B, S7E Fig). Whether images contained *Elephant* or *Pizzas* did not significantly affect accuracy for either subject (multivariate logistic regression, S2 Table), so they were pooled to create psychometric (Weibull) functions of response accuracy for each stimulation site (S7C, S7F Fig). Stimulation at any site or with any EVI did not significantly influence response accuracy (Chi-Square tests of independence, Bonferroni-corrected P > 0.05 across 75%, 50%, and 0% noise levels), even after adjusting for run number, trial timing within runs, image scene, and image coherence (multivariate logistic regression, S2 Table). The only robust predictor of response accuracy was image coherence (subject 1: *z* = 9.21, P = 3.4*10^-20^; subject 2: z = 7.55, P = 4.3*10^-14^).

#### Adjusted common average vs. bipolar re-referencing

In our evoked potential analysis, data were re-referenced by an adjusted common average that mitigated the inclusion of bias from other responsive channels (11). Local re-referencing methods, such as bipolar or Laplacian, were avoided because they can strongly distort or attenuate meaningful evoked potentials between neighboring electrodes (11). This can strongly confound interpretation if stimulation has no effect on the electrode of interest but evokes potentials at a neighboring electrode. However, highly focal signal features such as broadband changes might be better accentuated by local re-referencing (12). Thus, we also tested broadband analysis after bipolar re-referencing (electrodes 1-2 and 3-4 in S3 Fig).

Bipolar re-referencing reduced the peak amplitude of the stimulation broadband transients compared to adjusted common average re-referencing. This was likely attributable to better attenuation of sharp evoked potential peaks before filtering. This reduction was less pronounced in subject 1 than subject 2, and in neither case was the transient entirely eliminated. Therefore, the transients were unlikely to be filtering artifact alone.

Control stimulation in subject 1 modulated the visual induced broadband response when the data were bipolar re-referenced but not when adjusted common average re-referenced. (S3B Fig). This was the only case showing modulation of the visual broadband response. However, the increase in variance explained was marginal over the next best model, and the visual response time courses did not differ noticeably across stimulation conditions, so cautious interpretation is warranted. All other results were consistent regardless of reference choice.

### References

1. Tustison NJ, Avants BB, Cook PA, Zheng Y, Egan A, Yushkevich PA, et al. N4ITK: Improved N3 Bias Correction. IEEE Trans Med Imaging. 2010 Jun;29(6):1310–20. doi:10.1109/TMI.2010.2046908

2. Fonov V, Evans A, McKinstry R, Almli C, Collins D. Unbiased nonlinear average age-appropriate brain templates from birth to adulthood. NeuroImage. 2009 Jul 1;Organization for Human Brain Mapping 2009 Annual Meeting47:S102. doi:10.1016/S1053-8119(09)70884-5

3. Avants BB, Epstein CL, Grossman M, Gee JC. Symmetric diffeomorphic image registration with cross-correlation: Evaluating automated labeling of elderly and neurodegenerative brain. Med Image Anal. 2008 Feb 1;Special Issue on The Third International Workshop on Biomedical Image Registration – WBIR 200612(1):26–41. doi:10.1016/j.media.2007.06.004

4. Zhang Y, Brady M, Smith S. Segmentation of brain MR images through a hidden Markov random field model and the expectation-maximization algorithm. IEEE Trans Med Imaging. 2001 Jan;20(1):45–57. doi:10.1109/42.906424

5. Veraart J, Novikov DS, Christiaens D, Ades-aron B, Sijbers J, Fieremans E. Denoising of diffusion MRI using random matrix theory. NeuroImage. 2016 Nov 15;142:394–406. doi:10.1016/j.neuroimage.2016.08.016

6. Andersson JLR, Sotiropoulos SN. An integrated approach to correction for off-resonance effects and subject movement in diffusion MR imaging. NeuroImage. 2016 Jan 15;125:1063–78. doi:10.1016/j.neuroimage.2015.10.019

7. Andersson JLR, Graham MS, Zsoldos E, Sotiropoulos SN. Incorporating outlier detection and replacement into a non-parametric framework for movement and distortion correction of diffusion MR images. NeuroImage. 2016 Nov 1;141:556–72. doi:10.1016/j.neuroimage.2016.06.058

8. Matsumoto R, Nair DR, LaPresto E, Najm I, Bingaman W, Shibasaki H, et al. Functional connectivity in the human language system: a cortico-cortical evoked potential study. Brain. 2004 Oct;127(10):2316–30. doi:10.1093/brain/awh246

9. Miller KJ, Zanos S, Fetz EE, Nijs M den, Ojemann JG. Decoupling the Cortical Power Spectrum Reveals Real-Time Representation of Individual Finger Movements in Humans. J Neurosci. 2009 Mar 11;29(10):3132–7. doi:10.1523/JNEUROSCI.5506-08.2009 PubMed PMID: 19279250.

10. Winawer J, Kay KN, Foster BL, Rauschecker AM, Parvizi J, Wandell BA. Asynchronous broadband signals are the principal source of the BOLD response in human visual cortex. Curr Biol CB. 2013 Jul 8;23(13):1145–53. doi:10.1016/j.cub.2013.05.001 PubMed PMID: 23770184; PubMed Central PMCID: PMC3710543.

11. Huang H, Ojeda Valencia G, Gregg NM, Osman GM, Montoya MN, Worrell GA, et al. CARLA: Adjusted common average referencing for cortico-cortical evoked potential data. J Neurosci Methods. 2024 Jul 1;407:110153. doi:10.1016/j.jneumeth.2024.110153

12. Li G, Jiang S, Paraskevopoulou SE, Wang M, Xu Y, Wu Z, et al. Optimal referencing for stereo-electroencephalographic (SEEG) recordings. NeuroImage. 2018 Dec;183:327–35. doi:10.1016/j.neuroimage.2018.08.020
