# Supplementary material for: Single pulse electrical stimulation in white matter modulates iEEG visual responses in human early visual cortex
Source: PLoS Comput Biol. 2026 Jul 24;22(7):e1014563. doi: 10.1371/journal.pcbi.1014563 (PMC13426923; doi:10.1371/journal.pcbi.1014563)
Supplement: Attachment — Submitted filename: spesVisual_PLOSCB_response2reviewer_v2c.docx [file pcbi.1014563.s015.docx]

We thank the three reviewers for their time and constructive feedback. We have made changes to the manuscript based on their suggestions and provided responses to each comment individually, **in blue font below**. Wherever noted, line numbers correspond to those in the “clean”, unmarked copy of the revised manuscript. Please note that the supplemental text is now labeled “S13 Text” (previously “S12 Text”), and two new supplemental figures (S11 and S12 Figures) have been added.

**Reviewer #1**

General comments:

The ms. deals with an important and timely topic, namely the causal effects of brain stimulation on cortical function. In this case, the authors analyse the effects single electrical pulses to the white matter near the visual cortex. Using finite impulse response models, they authors assess the effects of the stimulation on two subjects during an ambiguous visual stimulation experiment.

We thank the reviewer for their recognition of the importance and timeliness of this work, as well as for the thoughtful comments that they have provided.

Specific comments:

1.1. The exact numerical procedures are somewhat ambiguous. The finite impulse models are fitted to the evoked potentials, but the necessary mathematical expressions are not supplied, in particular the dimensionality of the data to which the the models are fit. This lack is amplified by some figures showing apparent single trial data (Figure 1A) labelled 'evoked potentials' and some showing trial-averaged data (Figure 1C) with the same label. Similarly, the preprocessing section refers to evoked potentials, while presumably operating on the unaveraged time-series, then subsequent descriptions seems to refer to event-locked, trial-averaged evoked potentials, particularly when looking at the Figures (say, 4F&G).

We clarify that all modeling in our work is performed across single-trial evoked potential time series data to maximally incorporate its rich temporal dynamics and variability. We agree that this distinction is critical to the overall interpretation of the paper, so we have now taken care to clarify this in the relevant sections of the manuscript. Instances when trial-averaged data are shown are meant solely for visualization and reader interpretation. In the examples that the reviewer describes: Figure 1C is a schematic that illustrates what we might expect to see if non-additive modulation occurs – it is not data (neither single-trial nor average); in Figure 4F & G, the purpose of showing the trial average overlays is to serve as a visual reference to the reader for the quality of model fits, since a better fit should more closely approximate the trial mean. The single-trial time series data (to which the model was actually fitted) are present in gray. We have now revised the legends and captions in these figures for clarity.

The details and mathematical expressions of the finite impulse response models are provided in section 2.7, lines 217 - 265, which we have now revised carefully for clarity. We note here that all four FIR models are of the form y = Xβ + 𝜀, and then explain, in detail, what each variable represents.

REVISIONS:

- Clarifications added that modeling occurred on single-trial data. E.g., line 216: *“All modeling is performed on single-trial data”*, line 223: *“y is the concatenated vector of all observed single-trial evoked potentials”.*
- Figure 1C caption: clarified this is schematic and not depicting real data.
- Figure 4F, G: revised the in-figure legend (top right) to note single-trial data in gray.
- Revisions to section 2.7 (lines 217-231) and supplemental methods (S13 text, lines 72-87) to clarify mathematical equations of FIR models and to describe the dimensionality of data being modeled.
  - Dimensionality for FIR models on evoked potentials:

*“Across subjects and stimulation sites, t ranged from 129,923 to 287,633 (751 downsampled time points × 173–383 trials); and across the four FIR models (see below), p ranged from 1200 to 6000 (600 predicted time points × 2–10 sets of predictors).”*

- Dimensionality for FIR models on broadband changes:

*“The dimensionality was lower than for evoked potential data due to greater downsampling: t ranged from 52200 to 99963 (261 time points × 200–383 trials) and p ranged from 420 to 2100 (210 predicted time points × 2–10 sets of predictors).”*

1.2. Apart from the methodological ambiguity, two participants is simply insufficient to draw conclusions, even appreciating the difficulty of collecting this type of data in human subjects. If this were an animal study, with the same tracts carefully targeted, and analyses done at the single trial level so that each animal constitutes a test of the hypothesis, then it would still be reasonable to request one or two more animals. With the variability inherent in sEEG placement, guided by medical rationale, and apparent analysis of trial-averaged EPs, then many more participants would be required to draw conclusions.

Here, the reviewer brings up our low sample size of two participants. We understand that this is a valid concern and we agree that this sets a global limitation on our ability to generalize findings. We now discuss this limitation in much greater detail in the discussion section of the manuscript (see “REVISIONS” below). Here, we first describe our reasoning for why we believe that we have provided sufficient and robust evidence toward our conclusions.

First, all analyses were performed on single-trial data, as clarified for 1.1 above. Therefore, the within-subject validity afforded by the large number of replicates (~300 trials for each electrode) meant that each subject is itself a test of the hypothesis. For example, observe the narrow confidence intervals on model COD (e.g., Figure 4H, J), and the narrow confidence intervals on the coefficients themselves (e.g., Figures 4I, K, S2B), which are only possible with many trials and good signal-to-noise ratio.

Second, we emphasize the highly consistent anatomical targeting between the two subjects, with respect to the white matter tracts activated by the main stimulation sites (Figure 2A, D, top) and the locations of the primary measurement electrodes in the EVC (Figure 2B, E). This is further supported by the electrophysiological similarity in latencies and phases of main stimulation site BSEPs (e.g., Figure 2C top left vs. 2F top left): a rapid positive peak <20 ms, a prominent negative peak at 40 ms, and a slow positive phase which peaks ~100-150 ms. Although the anatomical targeting was not experimentally controlled, these observed similarities fortuitously allowed our study to approximate precise animal studies and to serve as further reinforcement for each participant as a test of the hypothesis. We speculate that we would obtain highly similar results if the same subjects were to return at a later date with the same electrode sites. In contrast, we also believe that the addition of other participants (with different electrode sites) would more likely confound our findings than contribute meaningfully to them. To this point, we have added a section in the discussion about our recommendations to other researchers who wish to robustly reproduce our findings.

REVISONS: Greatly expanded the discussion section regarding limitations imposed by sample size, such as the need for cautious interpretation about generalizability; and recommendations for future replication (lines 536-569).

This text now reads:

“*The experiments in this study were conducted in two young male adult humans. This limits the generalizability of our findings, and a cautious interpretation is advised. The iEEG electrodes were placed clinically and not optimized to be identical in both subjects, though the evidence provided by imaging supports general anatomical similarity in key electrode locations between our two subjects (Figure 2).*

*Diffusion MRI showed that the main stimulation site in each subject richly overlapped the same set of white matter tracts with projections to the visual cortex (Figure 2A and D, top). One caveat is that the relative composition of individual tracts is different between the two subjects: for example, CC-FM was more strongly stimulated in subject 1 than subject 2, while IFOF was more strongly stimulated in subject 2 than subject 1. Because stimulation simultaneously activated several major white matter tracts, we cannot presently pinpoint which pathway(s) might be chiefly responsible for the modulatory effects. Polysynaptic pathways indirectly linking the stimulation and measurement sites may also be involved.*

*Our primary measurement electrodes of interest (electrodes 1-4) were located in the early visual cortex (Figure 2B and E), based on segmentation with Freesurfer and the Benson probabilistic visual atlas. However, the visual stimulus captured by each electrode’s population receptive field likely differed since they were in different areas of the visual cortex. Nevertheless, stimulation had similar effects across the four electrodes and did not vary significantly by image. Of note, evoked potentials at electrodes 3 and 4 showed similar modulation (e.g., Figure 4J-K vs. S2A), despite them being located in V2 and V1, respectively. Overall, this supports a stimulation robustness that is not strongly dependent on precise retinotopy.*

*Despite differences in electrode placement and white matter activated, we discovered similar evoked potential shapes, broadband responses, and time interval-dependent modulation in both subjects. We emphasize that our results were well validated within each subject. Our single-trial analysis (with 6-24 trials per experimental condition and 173-383 trials per electrode) provided robust confidence intervals for all estimates of model coefficients and goodness-of-fit, and thus each subject served as an independent test of the hypothesis. In addition to testing the generalizability of these findings on more subjects, future studies could provide greatest benefit by systematically testing a range of stimulation and measurement sites. This could elucidate the extent of modulation throughout the rest of the visual system and determine which white matter tracts might be most implicated. We recommend a similar multimodal approach as ours that leverages CT, structural MRI, and diffusion MRI coregistration to precisely identify where electrodes are in relation to structural and functional brain areas, and in relation to major white matter tracts.*”

1.3. It would be more informative to analyze the effects of stimulation at the single trial level, since this is where the cortex itself operates. At the very least, any analysis should address the adequacy of aggregation over trials, e.g. amount of signal variance lost by this simple step.

We agree with the reviewer that aggregation over trials leads to significant information loss. As described in the response to 1.1, all of our modeling and analysis (e.g., even goodness-of-fit estimates like COD) were performed at the single-trial level. Figures depicting trial averages are intended for reader interpretability, and corresponding captions have now been clarified (e.g., for Figure 1C).

**Reviewer #2:**

General comments:

This article is an interesting and well-conducted study demonstrating how intracranial stimulation of white matter tracks, connected or not to visual areas, interact with visual evoked potentials in two epileptic patients. The article is clearly written, although I was sometimes a bit lost while reviewing a few methodological choices and result descriptions, and while navigating back and forth the main manuscript, the figures, and the supplementary materials. I will try to indicate where clarifications may be helpful. Overall, this is a solid work, which deserves publication, pending a few moderate revisions.

We thank the reviewer for their careful read of our article, and we have incorporated the suggestion to improve clarity.

Specific comments:

2.1. The introduction could more explicitly specify the technique (e.g., intracranial stimulation vs. TMS) from which knowledge has been gained.

We agree with the reviewer and have now clarified the introduction to specify neurostimulation techniques for each reference, with textual edits throughout the first paragraph (lines 54-68).

2.2. BSEPs: the acronym is used for the first time in Fig. 2 legend without explanation

We now correct this discrepancy by moving up the main text definitions for VEP and BSEP to lines 135-137, before Figure 1.

2.3. I was a bit confused with the “early visual measurement electrodes”, maybe “measurement electrodes within the early visual cortex” would be easier to read.

We agree with this recommendation for textual clarity.

REVISIONS:

- Changed all instances of “early visual measurement electrodes”, “EVC electrodes”, or similar combinations to “measurement electrodes in the EVC” throughout the manuscript. (e.g., in captions for S2 and S3 Figures)
- Similarly, changed “non-EVC electrodes” and similar phrases to “electrodes outside the EVC” throughout the manuscript (e.g., in S4 and S5 Figures).

2.4. I am a bit confused with the models’ definition, which is at the chore of the study.

We have revised the methods for clarity and tackle each of the sub-questions below in detail.

2.4.1. Not clear if SPES alone data is used to fit the models or not. If not, why?

The SPES-only (independent SPES) data was not used to fit the models. The SPES-only data was collected separately from the main SPES+Visual trials (i.e., not interleaved), and under different experimental circumstances (the patients were freely resting with no task instructions and did not fixate on a screen). These differences manifested as visibly different amplitudes between the SPES-only BSEPs and the SPES+Visual BSEPs, possibly related to differences in subject brain state (see response to 2.9 below for a new S11 Figure directly comparing the BSEPs). For these reasons, we believed it to be inappropriate to include the independent SPES data in the model.

REVISIONS:

- We clarify in section 2.4 that the independent SPES data are not used to fit the models (lines 147-149):

*“Since these data were collected under different experimental conditions, they were analyzed separately and not included in the main SPES+Visual analysis (Section 2.7).”*

2.4.2. Not sure when sham trials are used or not. And why do they have the same VEP predictors as the 200 ms EVI trials in the EVI model? They could have their own predictors, no?

To address when sham trials are used: All trials, including the sham trials and SPES+Visual trials, are always used when fitting all FIR models. All FIR models predict the same input data (all trials) and differ from one another only by specifying which factors are permitted to vary independently (none, EVI, image identity, or both). We explain this in lines 211-216:

“*These models test whether the observed responses are better explained by visual component responses that remain unchanged with stimulation (reflecting a pure additive relationship between stimulation and visual responses) or by visual component responses that change with varying EVI (reflecting an interaction between stimulation and visual responses). Simply put, for the same observed data, which independent predictors best explain the observed trial-to-trial differences?*”

To address why sham trials have the same VEP predictors as the 200 ms EVI trials in the EVI model, rather than their own predictors: The sham and 200 ms EVI conditions are merged for model fitting because the linear system with 4 stimulation conditions is constrained to fit at most 4 independent predictors (BSEP and 3 EVI-dependent VEPs). One way to conceptualize this is to treat the EVI FIR model as a linear system of 4 equations, which each correspond to a stimulation condition: sham, 0 ms, 100 ms, and 200 ms. This system allows for at most 4 unknowns (BSEP and 3 VEPs), which does not leave freedom for a fifth sham-only predictor. Solving for a sham-only predictor would be possible if another EVI were present in the experiment. We chose to merge the sham condition with the 200 ms EVI condition because 200 ms was the longest EVI and therefore most *temporally adjacent* to the sham stimulation condition. Therefore, their joint condition could be treated as “EVI of 200 ms *or longer*”.

We acknowledge that merging predictors for two stimulation conditions could result in some inaccuracy when solving for the underlying component evoked potential shapes, compared to a ground truth (e.g., the exact peaks and troughs in Figure 4D), and we now acknowledge this in the limitations section of the manuscript (lines 570-577). However, the primary finding that model accuracy significantly improves when it is allowed to vary by EVI should not be influenced by this merger. This supports our hypothesis that white matter stimulation modulates visual inputs in a time-dependent manner. In contrast, for control stimulation sites, model accuracy did not improve with the addition of EVI predictors, nor did the VEPs differ in shape across EVIs (S2B Figure).

REVISIONS

- Addition to lines 215-216 about goal of FIR modeling:

*“Simply put, for the same observed data, which independent predictors best explain the observed trial-to-trial differences?”*

- Additional clarifying text in the methods about the model design (lines 232-233):

*“The four FIR models include different sets of predictors in the design matrix, X, and their corresponding estimates in β. The input data, y, do not vary across models”.*

- Expanded on explanation about merging sham and 200 ms predictors (lines 249-252):

*“Because four stimulation conditions (sham and three EVIs) constrain the EVI model to a maximum of four independent sets of predictors (one BSEP and three EVI-dependent VEPs), sham trials were assigned the same VEP predictors as the 200 ms EVI condition (*$X_{VEP_{EVI200}}$*) to avoid an underdetermined system.”*

- Revised the legends in all figures in which 200 ms and sham trials were merged to say “EVI=200ms/sham” for clarity and transparency. This revision affected Figure 4 and S2-4 Figures.

2.4.3. I thought the matrices of Fig. 3 could help to clarify but actually they were more confusing to me. Below are a few examples of what I understood looking at them, which is probably wrong. I believe slightly expanding them and/or adding a bit more details in the models’ description would help to resolve unnecessary problems of interpretation.

• Simple model: SPES alone data not used to fit the model. Sham data used. Only two EVI used, simultaneous and 1 other (which one?). Nothing else, as there is no mention of extra lines or columns.

• Image model: SPES alone data used to fit the model (last row). Sham data used as well (middle row). Then, only the simultaneous EVI data is used (first row): I guess other EVIs are used too (with the same predictors) but it is not visible here.

• EVI model: I guess something is missing to show that there are more rows/columns to the matrix. I see that SPES alone is not used, sham is (second row), and I guess all the EVIs are used!

• Full model: If the intercept corresponds to the VEPs for 100% noise and 0-ms EVI, I do not understand why there is a shift of the intercept on the last row of the matrix.

We have now clarified that all SPES+Visual and sham trials are always used to fit the 4 FIR models (see response to 2.4.2 above).

To clarify the matrices in Figure 3 (and specifically the image and full models):

Regarding the image model (Figure 3B), the last row is not meant to depict SPES-only trials (which was not used for modeling as discussed in 2.4.1). In the last row, VEP predictors are hidden in the third column – the “...” depicts a condensing of 5 sets of predictors corresponding to images 2-6. We have now revised the figure so this condensing is more visually apparent.

Regarding the full model, the intercept term is present for all experimental conditions, not only for the 100% noise and 0-ms EVI experimental condition. It is the two-way factorial intercept. This means that it is the *only* VEP predictor present for the [100% noise, 0-ms EVI] condition (e.g., the first row in Figure 3D), while in all other experimental conditions that predictor would be present in addition to “deviation” predictors in VEP columns 2-9. In the last row of Figure 3D these deviation predictors are hidden in the condensed columns. Conceptually, we can represent this by subdividing the full linear system, *y = Xβ*, into an explicit 2-way factorial representation:

*y = SPES + intercept_100% noise, 0 EVI_ + X_deviations, other images_β_deviations, other images_ + X_deviations, other EVIs_β_deviations, other EVIs_* + 𝜀

We have now revised the methods to explicitly list out the subdivided linear system for each of the 4 models. The partially filled table below provides another angle for understanding the intercept term. It shows, for a few experimental conditions, which coefficients would be summed to yield the full model estimate.

|  | 100% noise | Elephant-75% noise | Elephant-50% noise | Elephant-0% noise | Pizzas- 75% noise | Pizzas-  50% noise | Pizzas-  0% noise |
| --- | --- | --- | --- | --- | --- | --- | --- |
| 0 ms | **BSEP +**  **Int** |  |  |  | BSEP +  Int + pizzas75 |  |  |
| 100 ms | BSEP +  Int + 100 ms |  |  |  | BSEP +  Int +  100 ms +  pizzas75 |  |  |
| 200 ms/Sham |  |  |  |  |  |  |  |

REVISIONS:

- Revised Figure 3 so that condensed columns in B and D are more visually apparent (gray background, more ellipses). We also point this out in the figure caption: “...” denotes multiple condensed predictor sets.
- Revised the methods descriptions for the 4 FIR models, now explicitly spelling out the linear system of equations for each model (lines 232-265). This now reads:

*“The four FIR models include different sets of predictors in the design matrix, X, and their corresponding estimates in β. The input data, y, do not vary across models. Here we separate X and β by predictor sets for each model, for clarity. The first and second FIR models assume that all observed evoked potentials are the sum of a BSEP and VEPs that do not change with stimulation:*

1. *For the simple model, X contains two sets of predictors: one set for the BSEP 0-1 s post-stimulation (*$X_{BSEP}$*), and one set for the VEP 0-1 s after visual onset (*$X_{VEP}$*), regardless of EVI or image identity (Figure 3A):*

$y=X_{BSEP}\beta_{BSEP}+X_{VEP}\beta_{VEP}+\varepsilon$*.*

2. *For the image model, X contains eight sets of predictors – one set for the BSEP (*$X_{BSEP}$*) and one set of VEP predictors for each image condition (*$X_{VEP_{img1-7}}$*) – which allow VEPs to vary by image (Figure 3B):*

$y=X_{BSEP}\beta_{BSEP}+X_{VEP_{img1}}\beta_{VEP_{img1}}+X_{VEP_{img2}}\beta_{VEP_{img2}}+X_{VEP_{img3}}\beta_{VEP_{img3}}+X_{VEP_{img4}}\beta_{VEP_{img4}}+X_{VEP_{img5}}\beta_{VEP_{img5}}+X_{VEP_{img6}}\beta_{VEP_{img6}}+X_{VEP_{img7}}\beta_{VEP_{img7}}+\varepsilon$*.*

*The third and fourth models allow visual responses to vary by stimulation condition:*

3. *For the EVI model, X contains four sets of predictors: one set for the BSEP (*$X_{BSEP}$*), and one set of VEP predictors for each EVI (*$X_{VEP_{EVI0}}$*,* $X_{VEP_{EVI100}}$*,* $X_{VEP_{EVI200}}$*) (Figure 3C). Because four stimulation conditions (sham and three EVIs) constrain the EVI model to a maximum of four independent sets of predictors (one BSEP and three EVI-dependent VEPs), sham trials were assigned the same VEP predictors as the 200 ms EVI condition (*$X_{VEP_{EVI200}}$*) to avoid an underdetermined system:*

$y=X_{BSEP}\beta_{BSEP}+X_{VEP_{EVI0}}\beta_{VEP_{EVI0}}+X_{VEP_{EVI100}}\beta_{VEP_{EVI100}}+X_{VEP_{EVI200}}\beta_{VEP_{EVI200}}+\varepsilon$*.*

4. *For the full model, X contains ten sets of predictors: one set for the BSEP (*$X_{BSEP}$*), and nine sets of VEP predictors in a two-way factorial design across image condition and EVI. The first set of VEP predictors serves as a two-way “intercept” term (*$X_{VEP_{0}}$*), which by itself represents the [0 ms EVI, 100% image noise] condition, and which is present on all experimental conditions. The next six sets (*$X_{VEP_{img2-7}}$*) encode deviations from the intercept to the other six image conditions, and the final two sets (*$X_{VEP_{EVI100}}, X_{VEP_{EVI200}}$*) encode deviations from the intercept to the 100 ms and 200ms/sham EVI conditions.*

$$y=X_{BSEP}\beta_{BSEP}+X_{VEP_{0}}\beta_{VEP_{0}}+$$

$$X_{VEP_{img2}}\beta_{VEP_{img2}}+X_{VEP_{img3}}\beta_{VEP_{img3}}+X_{VEP_{img4}}\beta_{VEP_{img4}}+X_{VEP_{img5}}\beta_{VEP_{img5}}+X_{VEP_{img6}}\beta_{VEP_{img6}}+X_{VEP_{img7}}\beta_{VEP_{img7}}+$$

$$X_{VEP_{EVI100}}\beta_{VEP_{EVI100}}+X_{VEP_{EVI200}}\beta_{VEP_{EVI200}}+\varepsilon.$$

Note the revised description for the full model which now provides greater clarity about the intercept term.

2.5. I was wondering if the split-half validation procedure is sufficient for the model selection, or if other methods, such as using the Akaike Information Criterion, could be used instead.

We did originally consider metrics such as AIC and BIC. Ultimately, we did not include them since they had parametric assumptions which are difficult to validate for our complex FIR models. We believe that the split-half validation procedure is sufficient and elegant because 1) it is nonparametric, transparent, and does not assume model structure; 2) it generates interpretable confidence intervals for each model; and 3) it is a well-established technique for model validation that controls for overfitting.

2.6. P. 13 “the earliest predictor time point (different for each trial)”: I don’t know what it is.

We now clarify the wording. The “earliest predictor time point” for a given trial refers to the first time point that a predictor occurs in the features matrix, *X*, for that trial, which depends on the stimulation condition. For example, if the trial has EVI = 200 ms, then *t_1_* for that trial is -200 ms relative to visual onset because that is when the predictors for the stimulation evoked potential component begin. This dynamic *t_1_* allows us to calculate COD using exactly all predicted time points.

REVISIONS: Rephrased this line (273-275):

“*t_1_ is the earliest predictor time point available based on stimulation condition (e.g., t_1_ = -0.2 s if EVI = 200 ms, t_1_ = 0 s if sham or EVI = 0 ms, relative to visual onset)*”

2.7. Concerning the psychometric analysis, I was surprised to find the description in the method and no results in the main manuscript. Either the results should be in the main ms, or the methods should go in the supplementary materials (and a brief mention could stay in the main ms).

REVISIONS:

We have now moved the psychometrics analysis methods to supplemental methods in S13 Text (lines 119-139). The section header (2.8 Psychometric Analysis) and a brief callout are kept in main text (lines 295-298).

2.8. Still concerning this psychometric, I appreciate the command of modelling of the authors, but why not simply perform on the RTs a 3-way ANOVA Image*Noise*EVI, separately for the two stimulation sites (unless there are good reasons to think the run number and trial onset time would completely hinder the detection of these effects). The choice of the two regression models used to test the impact on accuracy is not clear to me either.

The reviewer presents a reasonable alternative (the 3-way ANOVA) to our multivariate linear model on reaction time. Our rationale for choosing a multivariate linear regression that included run number and trial onset time as predictors was because we observed significant participant improvement/learning during the experiment (trial onset time for subject 1, run number for subject 2, S13 Text lines 156-158). If the adjustment variables were removed from the multivariate regression, then our categorical predictors in this ordinary least squares analysis would be directly equivalent to the independent factors in a multi-way ANOVA (e.g., [see this link](https://rinterested.github.io/statistics/ANOVA_is_regression.html)).

Regarding the reviewer’s second point (last sentence), we agree that having two separate logistic regression models to test accuracy was confusing. Our original intent was to use the first regression model to demonstrate that the scene identity did not significantly influence accuracy as support for pooling the two scenes to create the psychometric functions (S7C, F Figure). We then used the second regression model to adjust for image coherence while assessing the impact of stimulation on accuracy. Overall, this two-step approach was confusing, so we have now merged the two logistic regression models on accuracy into a single regression model and table (S10 Table).

REVISIONS:

- Merged the two previous logistic regression models on response accuracy into a single model (now S10 Table). This did not significantly affect any of the previous results. Image scene remains an insignificant predictor on response accuracy.
- Minor edits to table captions (S10 caption, lines 861-864), discussion (line 520), supplemental methods (S13 Text, lines 137-138), and supplemental results (S13 Text, lines 166-168) to be consistent with the merged model and to reflect the updated statistics.

2.9. “The BSEP components resembled the BSEPs recorded in the independent SPES task (Figure 2C), but with slightly greater amplitude” (p. 16). So here I interpret that the SPES data alone is not used to fit the models (see my interrogation at point 4.i above). If the SPES alone data is not used to fit the model, but as a way to compare the BSEPs components and the BSEPs recorded in the SPES alone task, maybe it would be nice to see them on a same plot for comparison?

As recommended, we now show them in the same plot for comparison and have added new S11 Figure comparing the BSEPs in the independent SPES task, the BSEP component predicted from the SPES+Visual task, as well as the observed SPES+Visual trials with 200 ms EVI (to allow for direct comparison between the observed data on the first 200 ms). This S11 Figure is now called out in the main text at line 338.

REVISIONS: New S11 Figure and caption, reproduced below.

**S11 Figure. Comparison of BSEPs from independent SPES task and from SPES+Visual task.** For each stimulation–measurement pair: observed BSEPs from the independent SPES task are shown in black (mean and 95% confidence interval), observed trials from the SPES+Visual task with EVI = 200 ms are shown in blue (mean and 95% confidence interval), and the stimulation evoked potential component predicted by the EVI model (from the SPES+Visual data) is shown in purple. The observed data in blue contain only stimulation effect on 0-200 ms, so it can be meaningfully compared to the other traces on this interval.

2.10. Similarly, I read in several place that the VEPs are modulated but it is difficult to evaluate how, because we never see the sham trials (and/or the sham component, if it was used in the EVI model) compared to the VEP components for all 3 EVIs. This is similar to the point 4.ii above. Given that BSEPs response/components are not over at 200 ms, I really do not understand why 200-ms EVI should have the same component than sham.

We hope we have mostly clarified the methodological necessity of the joint sham and 200-ms EVI predictors in response to 2.4.2. Here we clarify more on its interpretation. By assuming an identical component shape for the 200 ms EVI VEPs and sham VEPs, it is possible that we incur some degree of distortion in our estimates for the VEP shapes, distributed across stimulation conditions. We now discuss this limitation in greater detail in the manuscript. However, the evidence for VEP modulation lies primarily in the fact that the VEPs for the different stimulation conditions (sham + 200 ms vs. 100 ms vs. 0 ms) differ significantly from each other – and adding EVI as a degree of freedom to the FIR model significantly improves the goodness-of-fit (EVI model > simple model). In contrast, this is not the case for the control stimulation sites, where VEPs do not differ by EVI even when the BSEP is of large amplitude (S2B Figure). The predicted VEP components (along with their 95% confidence intervals) are compared directly to each other in Figures 4I, 4K, and S2. In these plots, the blue curves are predictions for the merged sham + 200 ms EVI trials, and they well approximate the observed sham trials (Figure 4A).

REVISIONS: Added discussion on the limitations of combining predictors for the 200 ms EVI and sham trials in the FIR model (lines 570-577):

“*Finally, as noted in the Methods, sham and 200 ms EVI trials were assigned the same VEP predictors to avoid an underdetermined FIR system. This may introduce some distortion in predicted component shapes, to the extent that the true sham and 200 ms VEPs differ. Nevertheless, this limitation is unlikely to undermine the primary evidence for VEP modulation, which rests on the significantly improved model goodness-of-fit when predictors were allowed to vary by stimulation condition. We recommend that future studies employ a greater diversity of EVIs to circumvent this limitation and more accurately constrain ground-truth component shapes.*”

2.11. Figure 4., it seems to be that Panel D and I represent the same thing (with confidence interval and stats in I and not D). Right?

This is correct. Reason for partial duplication: Panel D is meant to serve as one part of the 4-panel group B-E, which compares the predictions across the four FIR models (simple, image, EVI, full). Panel I goes into greater detail to show statistics and confidence intervals for the best-fitting model (EVI), which mirrors the subject 2 findings in Panel K.

REVISIONS:

- Added a box around Figure 4 panels B-E to better draw attention to their grouped nature.
- In Figure 4I caption, added “(D)” to clarify that the responses are the same:

“***I****, Bootstrapped mean and 95% confidence intervals for EVI model responses (D).*”

2.12. “SPES at the control stimulation sites did not modulate visual broadband responses either, and in both subjects it produces smaller stimulation-induced broadband response than stimulating in visual pathways (S3B Figure).” However, I found I the figure that the full model was best for the bipolar electrode 1-2. I was surprised to not find any comment on that here (until I found it in the S12).

The reviewer notes a valid contradiction here. This omission was due to the minimal relevance attributed to the isolated bipolar finding, but we have now revised to improve transparency and avoid confusion.

REVISIONS: Revised the section mentioned (Lines 382-388) to note the bipolar exception:

“*In general, SPES at the control stimulation sites did not modulate visual broadband responses either. The sole exception was one bipolar re-referencing case (S3B Figure, control stimulation to bipolar measurement electrodes 1-2), where the full model marginally outperformed the image model. The relevance of this isolated finding is uncertain. See S13 Text for more detail on bipolar re-referenced findings. Stimulating at control sites also produced smaller stimulation-induced broadband responses than stimulating in visual pathways (S3B Figure).*”

2.13. I am a bit confused with the model comparison. For example, I see the test performed “Full > EVI, EVI>Simple”. Sometimes “full > EVI/image and EVI/image > simple”. So I guess EVI and image are never directly compared? On the figures, some of the models are indicated as significantly different (*), some are not (ns), what about the other pairs, are there simply not compared? In that case, some pairs are compared for one electrode and not for another one. I struggle to find the logic.

We acknowledge that this process of model comparison may appear confusing at first glance. We limited statistical comparisons between certain models, instead of making all pairwise comparisons, to minimize the probability of committing type 1 (false positive) errors. The comparisons were made on the basis of hierarchical model complexity, and the goal was to determine which model *best* described the data, rather than generate a statistical ranking of all 4 models each time. This rationale is described in Section 2.7 (lines 277-288), which we have now revised for increased clarity.

An alternative way to understand this is by approaching backwards from the most to least complex model. If the full model has the highest mean COD, we compared it to the model with the next highest mean COD, and if this comparison was significant, then we stopped there: full model wins. If not, we turned to either the EVI or image model, whichever has higher mean COD, and compared that against the simple model. If this comparison was significant, then the EVI model or image model wins. The reviewer is correct in pointing out that the EVI and image models are never directly compared to each other, and this was intentional since they are on the same level of complexity (*+1 degree of freedom* over the simple model). If neither of the above comparisons were significant, the simple model wins. To summarize, this means a maximum of 2 comparisons are made per stimulation-measurement pair. Since all comparisons were right-tailed (H_a_: more complex model > less complex model), all trivial comparisons were omitted (i.e., 0 comparisons if simple model had highest mean COD, no comparisons against the full model if the full model did not have highest mean COD).

Figure 5 serves as a good example. In Figure 5A, we first checked whether the full model (with highest mean COD) significantly outperformed the next best model (EVI). This was not significant. Next, we checked whether the EVI model significantly outperformed the simple model, and this was also not significant, so the simple model was best. No comparisons were made against the image model because it had lower mean COD than the EVI model. In contrast, in Figure 5D, the full model did not have the highest mean COD so there were no trivial comparisons made against it. The image model (with higher mean COD than EVI model) significantly outperformed the lower complexity simple model and thus was deemed the best model.

REVISIONS: Revised the methods section (lines 277-288) to provide greater insight and clarity to the reader about the hierarchical comparison approach:

*“The models we designed are naturally ordered in terms of complexity, and we used split-half validated COD to determine whether any of the more complex models are necessary to explain the data. Whenever the simple model had the highest mean COD or no other model was significantly better, we concluded that the simple model best described the data. Whenever the higher-performing of the image or EVI models significantly outperformed the simple model (by paired t-test on COD, right-tailed P < 0.05) but was not significantly outperformed by the full model, we concluded that this model best described the data, indicating that EVI or image identity was a significant predictor of visual response. The image and EVI models were not directly compared to each other. Finally, whenever the full model significantly outperformed the next best model, we concluded that the full model best described the data, indicating that both EVI and image identity were significant predictors of visual response. This selective hierarchical comparison approach was employed to minimize the total type 1 error.”*

2.14. Concerning LOC electrodes: “Here, we observed a weaker but similar pattern of modulation as at EVC electrodes […]” (p. 20). I do not see the corresponding statistical results on Fig. S4C (and I still do not see the sham).

We have now added stats in S4C. We had originally omitted the statistics to avoid overcrowding the supplemental figure. But we agree that the statistical labels help support that claim in the main text.

The sham condition is combined with EVI = 200 ms here as discussed for the main analysis earlier (see response to 2.4.2). We have revised the legend here to say “200ms/sham”.

REVISIONS: Labeled the time intervals of significant difference between pairs of EVIs in S4C Figure (for Main stim – el-5 and Control stim – el-6).

2.15. About the statistical test with the Bootrapping procedure, I have not used such procedure myself, it sounds fair to me, but I was just surprised by the use of arbitrary boundaries for minimal length/number of points (20 ms for the comparison between components between them, 20% of the time for comparison against 0). How were these values chosen?

These chosen intervals were informed by domain knowledge rather than formal optimization.

The 20 ms bin size for comparing time intervals across EVI conditions was chosen because it approximates the duration of meaningful evoked potential features. For example, the N1 peak of the cortico-cortical evoked potential (our BSEP) is roughly 20 ms in duration (Matsumoto et al., 2004). At our downsampled rate of 800 Hz, this required 16 contiguous samples to be individually significant for the bin to be significant. A larger bin size would sacrifice temporal resolution and obscure rapid deflections like the N1, while a smaller bin size would increase noise and have minimal impact on interpretation when plotted.

For classifying significant stimulation responses, we required ≥20% of total time points within the 500 ms post-stimulation to be individually significant. The 500 ms window was chosen as the approximate upper bound for meaningful BSEP activity (e.g., Figure 2B, F), and it matches the end time of our COD calculations. The 20% threshold served as a practical cutoff, loosely inspired by the Pareto principle, to distinguish clearly prominent and longer-lasting stimulation components from weaker/transient ones. Since our goal was to identify a qualitative relationship between evoked potential and broadband modalities (Table 1), rather than make strict statistical conclusions, we believe that a lenient threshold was appropriate here.

REFERENCES:

Matsumoto, R., Nair, D. R., LaPresto, E., Najm, I., Bingaman, W., Shibasaki, H., & Lüders, H. O. (2004). Functional connectivity in the human language system: a cortico-cortical evoked potential study. *Brain, 127*(10), 2316-2330.

REVISIONS:

- Added explanatory text about these chosen values to the supplemental methods (S13 Text, lines 45-50):

*“20 ms was chosen because it approximates the duration of meaningful evoked potential features, such as the “N1” component in BSEPs (8). For the analysis in section 3.4, a stimulation response was considered significant only if ≥20% of total time points within the first 500 ms post-stimulation had 95% confidence intervals that excluded 0. These criteria assist in distinguishing clearly prominent and long-lasting stimulation responses from weaker/transient ones.”*

2.16. Concerning table 1 (which is a bit more confusing than clarifying to me).

2.16.1. Categorization of stimulation impact: Sentence such as “In contrast, stimulation-induced broadband changes did correlate with anatomical connectivity, as they were observed in EVC only when stimulating the main sites” (p. 15) is contradictory with Table 1 where Control->el1 and Control->el3 are in the second column: stimulation produced a significant broadband response.

The reviewer notes a valid discrepancy between the results reported at different parts of our analysis. We believe that this discrepancy arose primarily from minor differences in statistical power between the different data used. The quoted section from the main text (“ In contrast, … only when stimulating the main sites”) reports results from the independent SPES task (Figures 2C, F, and S1). This data contained 12 independent trials per stimulation condition, and was therefore less powered to detect subtle broadband changes from baseline. In contrast, the results in Table 1 (which match the plots in S3 Figure) were informed by FIR decomposition of hundreds of SPES+Visual trials, and therefore might be more sensitive to small but significant broadband changes from baseline. In both cases, the stimulation-induced broadband responses are much stronger with stimulation of main sites than control sites (e.g., S3A vs. B). In addition, some of the described discrepancy may be attributable to differences in experimental condition (resting vs. task-oriented), similar to the differences in BSEPs discussed in response 2.4.1 and 2.9 (S11 Figure).

Regardless of the reason for discrepancy, we agree that the current wording (i.e., “only”) was too absolute, and we have now tempered it.

REVISIONS: Minor textual revisions to lines 320-322:

“*stimulation-induced broadband changes did correlate with anatomical connectivity, as they were prominent in EVC when stimulating the main sites but generally weak or absent when stimulating the control sites*”.

This is now consistent with the Table 1 findings for control stimulation: small but significant stimulation-induced broadband changes at electrodes 1 and 3, but not at electrodes 2 and 4.

2.16.2. On Table 1, stimulation produce a significant VEP at Control-el2 and Control-el4, but I do not see it on Fig. S1 (at least for el2). In any case, there is not stats here (as it was the case for Fig. 2), so it is difficult to assess.

Similar to the discussion in 2.16.1 above, this discrepancy is due to differences between the SPES-only and SPES+Visual results. The significant control-el2 evoked potentials in Table 1 match the results in S2 Figure, which are FIR decompositions of the SPES+Visual dataset. In S2B, control-el1 and control-el2 have significant (95% CIs) but low-amplitude stimulation EPs. In contrast, Figures 2 and S1 show results from the independent SPES task which may differ from and have less precision than the SPES+Visual component estimates (see comparison in S11 Figure).

In Figures 2 and S1, we labeled significantly different time points (blue and red dots) only for the broadband estimates but not for the BSEPs because the BSEPs generally deviated from V=0, so many overhead red and blue dots would appear cluttered and not improve interpretation. However, we agree that for the weaker responses, it may be difficult to tell whether any time points deviated from 0. Since 95% confidence intervals are already included for the BSEPs, we now improve interpretation by adding horizontal V=0 lines.

REVISIONS: Added V=0 horizontal lines to the BSEPs in Figures 2C, F, and S1. Time points with 95% confidence intervals that do not overlap these lines are significant.

2.16.3. “SPES modulation of VEPs appears to be correlated with its success in inducing a stimulation broadband response” (and the preceding sentences, p. 20 – also in the discussion p. 24): what about the electrodes in the second row, second column (induce a significant broadband response but no modulation of VEP) or in the third row, first column (no significant broadband response but a significant modulation of VEP)?

In this section, our intention is to make observational claims on the patterns that we observed globally, rather than strict causal or statistical claims. Modulated VEPs *tended to overlap* with stimulation-induced broadband (row 3: more pairs in column 2 than column 1), whereas non-modulated VEPs *tended to overlap* with no stimulation-induced broadband (rows 1 & 2: more pairs in column 1 than column 2). We acknowledge that the pattern is not perfect (i.e., some pairs exist in [2,2] and [3,1]), as the reviewer notes.

REVISIONS:

- In the results section (lines 420-424): we tempered the language and avoided strictly statistical language (e.g., *“more likely to induce”, “roughly overlap”*):

“*This shows that SPES which modulated VEPs was also more likely to induce a stimulation broadband response (Table 1, third row, column 2 > column 1), whereas SPES which failed to modulate VEPs was more likely to induce no broadband response (Table 1, second row, column 1 > column 2). Thus, SPES modulation of VEPs appears to roughly overlap with its ability to induce a stimulation broadband response.*”

- In the discussion (lines 481-485): similar revisions:

“*This general pattern emerged at the group level across all visually responsive measurement electrodes in and out of the EVC: SPES that modulated the VEP more often than not produced a broadband response (Table 1, third row, column 2 > column 1), while SPES that did not modulate the VEP more often than not did not produce a broadband response (Table 1, second row, column 1 > column 2).*”

2.17. In the discussion: “Interestingly, this time course is comparable to that of intracortical facilitation seen in paired pulse stimulation experiments with transcranial magnetic stimulation and intracranial stimulation” (p. 22): 100 ms is within the inhibition window in paired-pulse paradigms.

Our original phrasing here was ambiguous, which led to miscommunication. We agree that 100 ms is within the intracortical inhibition window, and we meant to highlight that shorter paired pulse ISIs (e.g., 50 ms) = more facilitatory, which was analogous to the finding in our study that short EVIs (especially EVI=0 ms) led to significant VEP facilitation. We have now rephrased this section to be more specific.

REVISIONS: Rephrased lines 446-451:

“*The timing of the single pulse was important, as EVIs of 0 and 100 ms had distinct modulatory effects on the VEP. This parallels paired-pulse transcranial magnetic stimulation and intracranial stimulation findings, where the effect of a conditioning stimulus on a test stimulus depends too on the interstimulus interval – facilitation when ≤50 ms but inhibition when ≥100 ms (37). Similar mechanisms might underlie these paired-pulse findings as ours”.*

2.18. “suppression of ongoing activity is a more likely explanation” (p. 23). What about desynchronization?

This is an interesting point of discussion. Desynchronization (in the context of event-related desynchronization, ERD) typically refers to decreased power in alpha and beta bands, reflecting reduced low-frequency synchrony across the measured neuronal population. ERD usually occurs with increased cortical activity and is concurrent with *increased* broadband power. The decreased alpha/beta power during desynchronization relies on the fact that low frequency power is largely driven by synchrony in specific oscillatory bands. In contrast, broadband power is not a measurement of oscillatory amplitude, but rather driven by asynchronous, aperiodic, neuronal spikes (e.g., Miller et al., 2009). Therefore, broadband power decreases are unlikely to be due to further “desynchronization” of any oscillation. We respectfully maintain that a suppression of ongoing activity (neuronal firing) is a more plausible and general explanation for superimposed decreases in broadband power.

REFERENCES:

Miller, K. J., Sorensen, L. B., Ojemann, J. G., & Den Nijs, M. (2009). Power-law scaling in the brain surface electric potential. *PLoS computational biology, 5*(12).

REVISIONS: We clarify in the text (line 465) that we indicate a suppression of broadband activity:

“*Rather, suppression of ongoing broadband activity is a more likely explanation (17).*”

2.19. “Visual modulation” (subtitle 4.3, p. 23, and 4.4, p. 24). To be replaced by “visual evoked potential modulation”

We agree with and have made these suggested revisions.

2.20. S6 Figure not called in the main manuscript.

We have now added a main manuscript callout. Previously, it was only called out in the supplemental methods (S13 Text).

REVISIONS: Added callout for S6 Figure in the main text methods, when referencing the supplemental methods for FIR broadband modeling (lines 218-220):

“*The FIR analysis for broadband responses followed a similar procedure with a few key differences (see supplemental methods in S13 Text and S6 Figure for details).*”

2.21. S10 Table. Why “coherence:Elephant”?

This was the interaction term between image coherence and scene identity in the old logistic regression model (now deleted in response to 2.8). The naming convention served to indicate that the coefficient sign was positive when the scene was *Elephant* (and negative for *Pizzas*). In the current merged logistic regression model (S10 Table), this term is no longer present. We omitted it because it did not add much to the interpretation. The current regression model only keeps the main effects, *Elephant* vs. *Pizzas* and image coherence.

**Reviewer #3**

General comments:

In this manuscript, the authors use single pulse stimulation through SEEG electrodes placed in white matter tracks that connect with early visual cortex to examine how this stimulation modulates the neural response in early visual cortex, both to the stimulation alone and how it modulates the neural activity in response to visual stimuli presentation. This work sheds light on questions of whether electrical stimulation is merely additive to sensory input or whether it modulates how the neurons respond to that input. The authors use a rigorous model based approach to analyzing the neural signals to addressing this question. The one caveat is that this work was done in just 2 participants, which, on one hand, given the difficulty of finding patients with electrodes implanted in the same white matter tracks that feed the same early visual regions is understandable, it does limit the generalizability of the findings. The authors acknowledge this limitation in the results, though perhaps this caveat could be featured more prominently. Two participants is also comparable to non-human primate studies, though the norm in iEEG has become substantially more than that.

Overall, this manuscript is well written, the methods strong, and the results clear. I just have a few suggestions that may add to the contribution of the work and clarity.

We thank the reviewer for their careful reading and thoughtful suggestions. We have made revisions in line with their suggestions.

Specific (major) comment:

3.1. I would suggest adding a time-frequency type analysis to the work as this would add substantial richness to understand what aspects of the signals are additive versus modulatory. Providing more richness than the VEP and broadband by including a frequency domain analysis would add to the potential impact of the work.

We agree with the reviewer that the addition of a time-frequency analysis could help to add richness and robustness to this work. We have now included a time-frequency analysis in the revised manuscript for the primary main and control stimulation-measurement electrode pairs in the EVC. We did not explicitly model the time-frequency decompositions using FIR analysis, however. This is because the increased dimensionality of time-frequency data and the worse signal-to-noise ratio at individual frequency bins (compared to broadband) would significantly decrease the reliability and interpretability of model outputs.

Overall, our time-frequency analysis provides additional support for superimposed induced visual and electrical broadband changes. Visual-induced broadband increases were also accompanied by alpha band (8-12 Hz) decreases, which are expected and which reflect cortical disinhibition (e.g., Jensen et al., 2010). Lastly, we observe some curious electrical stimulation-induced power increase in the 20-50 Hz range, which matches spectral changes in our earlier SPES work (Huang et al., 2023). However, this finding was not consistent across all conditions.

REFERENCES:

Huang, H., Gregg, N. M., Ojeda Valencia, G., Brinkmann, B. H., Lundstrom, B. N., Worrell, G. A., ... & Hermes, D. (2023). Electrical stimulation of temporal and limbic circuitry produces distinct responses in human ventral temporal cortex. *The Journal of Neuroscience*, *43*(24), 4434-4447.

Jensen, O., & Mazaheri, A. (2010). Shaping functional architecture by oscillatory alpha activity: gating by inhibition. *Frontiers in human neuroscience*, *4*, 186.

REVISIONS:

- New supplemental figure (S12 Figure) which presents the result of the time-frequency analysis, reproduced below.
- New supplemental methods section “Time-frequency analysis” in S13 Text (lines 102-118), with a brief callout in the main text (lines 205-206):

“*Time-frequency analysis was performed using continuous wavelet transformation of the single-trial evoked potential data. See details in supplemental methods (S13 Text)*.”

- Results of the time-frequency analysis described in section 3.1 (lines 324-327):

“*Time-frequency analysis of the data confirmed these stimulation-induced broadband changes, and showed possible increases in low gamma range power (~20-50 Hz) when stimulating the main sites (S12 Figure).*”

and in section 3.3 (lines 390-399):

“*Time-frequency analysis of the data, in the form of wavelet spectrograms, largely recapitulated these broadband effects of electrical stimulation on visual processing (S12 Figure). Visual responses were characterized by a transient increase in broadband power that tapered over time, accompanied by a sustained decrease in alpha band (8-12 Hz) power. At the main stimulation sites, electrical stimulation produced a superimposed transient broadband increase followed by prolonged suppression, visible in the t-statistic heatmaps comparing each EVI condition to sham. This suppression was less consistent across control stimulation sites. Stimulation at the main site in subject 1 additionally produced increased power in the low gamma range (~20-50 Hz), consistent with the time-frequency findings from the independent SPES data.*”

**S12 Figure. Time-frequency analysis of SPES and visual responses.** For each stimulation–measurement pair: (Left) Average wavelet spectrogram for each stimulation condition of the SPES+Visual task, time-locked to visual onset. (Top right) Average wavelet spectrogram for the independent SPES task. For comparison, the broadband response from Figure 2C, F is copied above. (Bottom right) t-statistic heatmaps comparing each SPES+Visual EVI to sham, time-locked to stimulation. These heatmaps estimate the time-frequency effect attributable to SPES in the SPES+Visual task (i.e., stimulation component). For comparison, the broadband FIR stimulation component from Figures 5B, E, or S3B is copied above.

Specific (minor) comments:

3.2. It took me quite some time to understand why different analyses are shown for the two subjects in figure 5/why the right and left side of the figure were not parallel to each other. While I did figure it out in the end, it would be helpful to be explicit as to why different results are shown in two sides of this figure. A sentence or two in the figure legend making this explicit, rather than leaving it to the reader to figure this out, would greatly help clarity for the reader.

We appreciate the reviewer’s care in working through this figure. Yes, the right and left sides of the figure are different because they contrast the best-fitting FIR model in each subject (simple model in subject 1, image model in subject 2). We have now added text in the figure and in the figure caption to assist with interpretation.

REVISIONS:

- Revisions in Figure 5: Above panels B and E, the label now reads *“Best model = (simple/image) model”*. We also added text boxes on the left and right sides of the figure to point out to the difference: *“visual response independent of EVI or image noise”*, and *“visual response depends on image noise”*
- Clarifying revisions in the Figure 5 caption: *“the best fit (simple/image) model”* when describing B and E. Addition of a sentence: *“The simple model predictions provide a poorer fit to the data means than the image model predictions, when varying image noise.”*

3.3. More extensive discussion of the limitations from having only two participants is warranted. In particular, it would be helpful to provide a discussion of the what was quantitatively and qualitatively different between the participants to give some indications of what between subject variability needs to be examined in future studies.

We agree with the reviewer that a more extensive discussion of this limitation is warranted. We have now significantly expanded the discussion section with respect to limitations imposed by sample size, lines 536-569 (see response to comment 1.2). In the limitations section, we now discuss similarities and differences between the two subjects, such as the different ratios of white matter tracts involved and variable placement of measurement electrodes in the early visual cortex. We also provide brief recommendations for future studies wishing to replicate and expand upon our findings.
