# Supplementary material for: Single pulse electrical stimulation in white matter modulates iEEG visual responses in human early visual cortex
Source: PLoS Comput Biol. 2026 Jul 24;22(7):e1014563. doi: 10.1371/journal.pcbi.1014563 (PMC13426923; doi:10.1371/journal.pcbi.1014563)
Supplement: Peer review history

# Peer review: Decision Letter 0

**DOI:** 10.1371/journal.pcbi.1014563.r001

**Authors:** Daniele Marinazzo, Laura Dugué

14 Jan 2026

PCOMPBIOL-D-25-01303

Single pulse electrical stimulation in white matter modulates iEEG visual responses in human early visual cortex

PLOS Computational Biology

Dear Dr. Huang,

Thank you for submitting your manuscript to PLOS Computational Biology. After careful consideration, we feel that it has merit but does not fully meet PLOS Computational Biology's publication criteria as it currently stands. Therefore, we invite you to submit a revised version of the manuscript that addresses the points raised during the review process.

Please submit your revised manuscript by Mar 16 2026 11:59PM. If you will need more time than this to complete your revisions, please reply to this message or contact the journal office at ploscompbiol@plos.org. When you're ready to submit your revision, log on to https://www.editorialmanager.com/pcompbiol/ and select the 'Submissions Needing Revision' folder to locate your manuscript file.

Please include the following items when submitting your revised manuscript:

* A letter that responds to each point raised by the editor and reviewer(s). You should upload this letter as a separate file labeled 'Response to Reviewers'. This file does not need to include responses to formatting updates and technical items listed in the 'Journal Requirements' section below.

* A marked-up copy of your manuscript that highlights changes made to the original version. You should upload this as a separate file labeled 'Revised Manuscript with Track Changes'.

* An unmarked version of your revised paper without tracked changes. You should upload this as a separate file labeled 'Manuscript'.

If you would like to make changes to your financial disclosure, competing interests statement, or data availability statement, please make these updates within the submission form at the time of resubmission. Guidelines for resubmitting your figure files are available below the reviewer comments at the end of this letter

We look forward to receiving your revised manuscript.

Kind regards,

Laura Dugué

Academic Editor

PLOS Computational Biology

Daniele Marinazzo

Section Editor

PLOS Computational Biology

**Additional Editor Comments:**

Reviewers' opinion diverge, with one reviewer pointing out substantial concerns. Please pay careful attention to all reviewers' comments.

**Journal Requirements:**

1) Please upload all main figures as separate Figure files in .tif or .eps format. For more information about how to convert and format your figure files please see our guidelines:

https://journals.plos.org/ploscompbiol/s/figures

2) Some material included in your submission may be copyrighted. According to PLOSu2019s copyright policy, authors who use figures or other material (e.g., graphics, clipart, maps) from another author or copyright holder must demonstrate or obtain permission to publish this material under the Creative Commons Attribution 4.0 International (CC BY 4.0) License used by PLOS journals. Please closely review the details of PLOSu2019s copyright requirements here: PLOS Licenses and Copyright. If you need to request permissions from a copyright holder, you may use PLOS's Copyright Content Permission form.

Please respond directly to this email and provide any known details concerning your material's license terms and permissions required for reuse, even if you have not yet obtained copyright permissions or are unsure of your material's copyright compatibility. Once you have responded and addressed all other outstanding technical requirements, you may resubmit your manuscript within Editorial Manager.

Potential Copyright Issues:

- Figure 1.. Please confirm whether you drew the images / clip-art within the figure panels by hand. If you did not draw the images, please provide (a) a link to the source of the images or icons and their license / terms of use; or (b) written permission from the copyright holder to publish the images or icons under our CC BY 4.0 license. Alternatively, you may replace the images with open source alternatives. See these open source resources you may use to replace images / clip-art:

- https://commons.wikimedia.org

- https://openclipart.org/.

**Reviewers' comments:**

Reviewer's Responses to Questions

**Comments to the Authors:**

**Please note here if the review is uploaded as an attachment.**

Reviewer #1: The ms. deals with an important and timely topic, namely the causal effects of brain stimulation on cortical function. In this case, the authors analyse the effects single electrical pulses to the white matter near the visual cortex. Using finite impulse response models, they authors assess the effects of the stimulation on two subjects during an ambiguous visual stimulation experiment.

The exact numerical procedures are somewhat ambiguous. The finite impulse models are fitted to the evoked potentials, but the necessary mathematical expressions are not supplied, in particular the dimensionality of the data to which the the models are fit. This lack is amplified by some figures showing apparent single trial data (Figure 1A) labelled 'evoked potentials' and some showing trial-averaged data (Figure 1C) with the same label. Similarly, the preprocessing section refers to evoked potentials, while presumably operating on the unaveraged time-series, then subsequent descriptions seems to refer to event-locked, trial-averaged evoked potentials, particularly when looking at the Figures (say, 4F&G).

Apart from the methodological ambiguity, two participants is simply insufficient to draw conclusions, even appreciating the difficulty of collecting this type of data in human subjects. If this were an animal study, with the same tracts carefully targetted, and analyses done at the single trial level so that each animal constitutes a test of the hypothesis, then it would still be reasonable to request one or two more animals. With the variability inherent in sEEG placement, guided by medical rationale, and apparent analysis of trial-averaged EPs, then many more participants would be required to draw conclusions.

It would be more informative to analyze the effects of stimulation at the single trial level, since this is where the cortex itself operates. At the very least, any analysis should address the adeqacy of aggregation over trials, e.g. amount of signal variance lost by this simple step.

Reviewer #2: This article is an interesting and well-conducted study demonstrating how intracranial stimulation of white matter tracks, connected or not to visual areas, interact with visual evoked potentials in two epileptic patients. The article is clearly written, although I was sometimes a bit lost while reviewing a few methodological choices and result descriptions, and while navigating back and forth the main manuscript, the figures, and the supplementary materials. I will try to indicate where clarifications may be helpful. Overall, this is a solid work, which deserves publication, pending a few moderate revisions.

1. The introduction could more explicitly specify the technique (e.g., intracranial stimulation vs. TMS) from which knowledge has been gained.

2. BSEPs: the acronym is used for the first time in Fig. 2 legend without explanation

3. I was a bit confused with the “early visual measurement electrodes”, maybe “measurement electrodes within the early visual cortex” would be easier to read.

4. I am a bit confused with the models’ definition, which is at the chore of the study.

i. Not clear if SPES alone data is used to fit the models or not. If not, why?

ii. Not sure when sham trials are used or not. And why do they have the same VEP predictors as the 200 ms EVI trials in the EVI model? They could have their own predictors, no?

iii. I thought the matrices of Fig. 3 could help to clarify but actually they were more confusing to me. Below are a few examples of what I understood looking at them, which is probably wrong. I believe slightly expanding them and/or adding a bit more details in the models’ description would help to resolve unnecessary problems of interpretation.

• Simple model: SPES alone data not used to fit the model. Sham data used. Only two EVI used, simultaneous and 1 other (which one?). Nothing else, as there is no mention of extra lines or columns.

• Image model: SPES alone data used to fit the model (last row). Sham data used as well (middle row). Then, only the simultaneous EVI data is used (first row): I guess other EVIs are used too (with the same predictors) but it is not visible here.

• EVI model: I guess something is missing to show that there are more rows/columns to the matrix. I see that SPES alone is not used, sham is (second row), and I guess all the EVIs are used!

• Full model: If the intercept corresponds to the VEPs for 100% noise and 0-ms EVI, I do not understand why there is a shift of the intercept on the last row of the matrix.

5. I was wondering if the split-half validation procedure is sufficient for the model selection, or if other methods, such as using the Akaike Information Criterion, could be used instead.

6. P. 13 “the earliest predictor time point (different for each trial)”: I don’t know what it is.

7. Concerning the psychometric analysis, I was surprised to find the description in the method and no results in the main manuscript. Either the results should be in the main ms, or the methods should go in the supplementary materials (and a brief mention could stay in the main ms).

8. Still concerning this psychometric, I appreciate the command of modelling of the authors, but why not simply perform on the RTs a 3-way ANOVA Image*Noise*EVI, separately for the two stimulation sites (unless there are good reasons to think the run number and trial onset time would completely hinder the detection of these effects). The choice of the two regression models used to test the impact on accuracy is not clear to me either.

9. “The BSEP components resembled the BSEPs recorded in the independent SPES task (Figure 2C), but with slightly greater amplitude” (p. 16). So here I interpret that the SPES data alone is not used to fit the models (see my interrogation at point 4.i above). If the SPES alone data is not used to fit the model, but as a way to compare the BSEPs components and the BSEPs recorded in the SPES alone task, maybe it would be nice to see them on a same plot for comparison?

10. Similarly, I read in several place that the VEPs are modulated but it is difficult to evaluate how, because we never see the sham trials (and/or the sham component, if it was used in the EVI model) compared to the VEP components for all 3 EVIs. This is similar to the point 4.ii above. Given that BSEPs response/components are not over at 200 ms, I really do not understand why 200-ms EVI should have the same component than sham.

11. Figure 4., it seems to be that Panel D and I represent the same thing (with confidence interval and stats in I and not D). Right?

12. “SPES at the control stimulation sites did not modulate visual broadband responses either, and in both subjects it produces smaller stimulation-induced broadband response than stimulating in visual pathways (S3B Figure).” However, I found I the figure that the full model was best for the bipolar electrode 1-2. I was surprised to not find any comment on that here (until I found it in the S12).

13. I am a bit confused with the model comparison. For example, I see the test performed “Full > EVI, EVI>Simple”. Sometimes “full > EVI/image and EVI/image > simple”. So I guess EVI and image are never directly compared? On the figures, some of the models are indicated as significantly different (*), some are not (ns), what about the other pairs, are there simply not compared? In that case, some pairs are compared for one electrode and not for another one. I struggle to find the logic.

14. Concerning LOC electrodes: “Here, we observed a weaker but similar pattern of modulation as at EVC electrodes […]” (p. 20). I do not see the corresponding statistical results on Fig. S4C (and I still do not see the sham).

15. About the statistical test with the Bootrapping procedure, I have not used such procedure myself, it sounds fair to me, but I was just surprised by the use of arbitrary boundaries for minimal length/number of points (20 ms for the comparison between components between them, 20% of the time for comparison against 0). How were these values chosen?

16. Concerning table 1 (which is a bit more confusing than clarifying to me).

i. Categorization of stimulation impact: Sentence such as “In contrast, stimulation-induced broadband changes did correlate with anatomical connectivity, as they were observed in EVC only when stimulating the main sites” (p. 15) is contradictory with Table 1 where Control->el1 and Control->el3 are in the second column: stimulation produced a significant broadband response.

ii. On Table 1, stimulation produce a significant VEP at Control-el2 and Control-el4, but I do not see it on Fig. S1 (at least for el2). In any case, there is not stats here (as it was the case for Fig. 2), so it is difficult to assess.

iii. “SPES modulation of VEPs appears to be correlated with its success in inducing a stimulation broadband response” (and the preceding sentences, p. 20 – also in the discussion p. 24): what about the electrodes in the second row, second column (induce a significant broadband response but no modulation of VEP) or in the third row, first column (no significant broadband response but a significant modulation of VEP)?

17. In the discussion: “Interestingly, this time course is comparable to that of intracortical facilitation seen in paired pulse stimulation experiments with transcranial magnetic stimulation and intracranial stimulation” (p. 22): 100 ms is within the inhibition window in paired-pulse paradigms.

18. “suppression of ongoing activity is a more likely explanation” (p. 23). What about desynchronization?

19. “Visual modulation” (subtitle 4.3, p. 23, and 4.4, p. 24). To be replaced by “visual evoked potential modulation”

20. S6 Figure not called in the main manuscript.

21. S10 Table. Why “coherence:Elephant”?

Reviewer #3: In this manuscript, the authors use single pulse stimulation through SEEG electrodes placed in white matter tracks that connect with early visual cortex to examine how this stimulation modulates the neural response in early visual cortex, both to the stimulation alone and how it modulates the neural activity in response to visual stimuli presentation. This work sheds light on questions of whether electrical stimulation is merely additive to sensory input or whether it modulates how the neurons respond to that input. The authors use a rigorous model based approach to analyzing the neural signals to addressing this question. The one caveat is that this work was done in just 2 participants, which, on one hand, given the difficulty of finding patients with electrodes implanted in the same white matter tracks that feed the same early visual regions is understandable, it does limit the generalizability of the findings. The authors acknowledge this limitation in the results, though perhaps this caveat could be featured more prominently. Two participants is also comparable to non-human primate studies, though the norm in iEEG has become substantially more than that.

Overall, this manuscript is well written, the methods strong, and the results clear. I just have a few suggestions that may add to the contribution of the work and clarity.

Substantive suggestion

• I would suggest adding a time-frequency type analysis to the work as this would add substantial richness to understand what aspects of the signals are additive versus modulatory. Providing more richness than the VEP and broadband by including a frequency domain analysis would add to the potential impact of the work.

Minor points

• It took me quite some time to understand why different analyses are shown for the two subjects in figure 5/why the right and left side of the figure were not parallel to each other. While I did figure it out in the end, it would be helpful to be explicit as to why different results are shown in two sides of this figure. A sentence or two in the figure legend making this explicit, rather than leaving it to the reader to figure this out, would greatly help clarity for the reader.

• More extensive discussion of the limitations from having only two participants is warranted. In particular, it would be helpful to provide a discussion of the what was quantitatively and qualitatively different between the participants to give some indications of what between subject variability needs to be examined in future studies.

**********

**Have the authors made all data and (if applicable) computational code underlying the findings in their manuscript fully available?**

The PLOS Data policy requires authors to make all data and code underlying the findings described in their manuscript fully available without restriction, with rare exception (please refer to the Data Availability Statement in the manuscript PDF file). The data and code should be provided as part of the manuscript or its supporting information, or deposited to a public repository. For example, in addition to summary statistics, the data points behind means, medians and variance measures should be available. If there are restrictions on publicly sharing data or code —e.g. participant privacy or use of data from a third party—those must be specified.

Reviewer #1: Yes

Reviewer #2: Yes

Reviewer #3: None

**********

PLOS authors have the option to publish the peer review history of their article (what does this mean?). If published, this will include your full peer review and any attached files.

If you choose “no”, your identity will remain anonymous but your review may still be made public.

**Do you want your identity to be public for this peer review?**  For information about this choice, including consent withdrawal, please see our Privacy Policy.

Reviewer #1: No

Reviewer #2: No

Reviewer #3: No

[NOTE: If reviewer comments were submitted as an attachment file, they will be attached to this email and accessible via the submission site. Please log into your account, locate the manuscript record, and check for the action link "View Attachments". If this link does not appear, there are no attachment files.]

**Figure resubmission:**

While revising your submission, we strongly recommend that you use PLOS’s NAAS tool (https://ngplosjournals.pagemajik.ai/artanalysis) to test your figure files. NAAS can convert your figure files to the TIFF file type and meet basic requirements (such as print size, resolution), or provide you with a report on issues that do not meet our requirements and that NAAS cannot fix.

After uploading your figures to PLOS’s NAAS tool - https://ngplosjournals.pagemajik.ai/artanalysis, NAAS will process the files provided and display the results in the "Uploaded Files" section of the page as the processing is complete. If the uploaded figures meet our requirements (or NAAS is able to fix the files to meet our requirements), the figure will be marked as "fixed" above. If NAAS is unable to fix the files, a red "failed" label will appear above. When NAAS has confirmed that the figure files meet our requirements, please download the file via the download option, and include these NAAS processed figure files when submitting your revised manuscript.

**Reproducibility:**

To enhance the reproducibility of your results, we recommend that authors of applicable studies deposit laboratory protocols in protocols.io, where a protocol can be assigned its own identifier (DOI) such that it can be cited independently in the future. Additionally, PLOS ONE offers an option to publish peer-reviewed clinical study protocols. Read more information on sharing protocols at https://plos.org/protocols?utm_medium=editorial-email&utm_source=authorletters&utm_campaign=protocols

# Author comment: Author response to Decision Letter 1

**DOI:** 10.1371/journal.pcbi.1014563.r002

9 May 2026

Attachment Submitted filename: spesVisual_PLOSCB_response2reviewer_v2c.docx

# Peer review: Decision Letter 1

**DOI:** 10.1371/journal.pcbi.1014563.r003

**Authors:** Daniele Marinazzo, Laura Dugué

9 Jul 2026

Dear Mr. Huang,

We are pleased to inform you that your manuscript 'Single pulse electrical stimulation in white matter modulates iEEG visual responses in human early visual cortex' has been provisionally accepted for publication in PLOS Computational Biology.

Before your manuscript can be formally accepted you will need to complete some formatting changes, which you will receive in a follow up email. A member of our team will be in touch with a set of requests.

Please note that your manuscript will not be scheduled for publication until you have made the required changes, so a swift response is appreciated.

IMPORTANT: The editorial review process is now complete. PLOS will only permit corrections to spelling, formatting or significant scientific errors from this point onwards. Requests for major changes, or any which affect the scientific understanding of your work, will cause delays to the publication date of your manuscript.

Should you, your institution's press office or the journal office choose to press release your paper, you will automatically be opted out of early publication. We ask that you notify us now if you or your institution is planning to press release the article. All press must be co-ordinated with PLOS.

Thank you again for supporting Open Access publishing; we are looking forward to publishing your work in PLOS Computational Biology.

Best regards,

Laura Dugué

Academic Editor

PLOS Computational Biology

Daniele Marinazzo

Section Editor

PLOS Computational Biology

***********************************************************

At the production process, please add a sentence in section S13 on the type of wavelet and the corresponding parameters.

Reviewer's Responses to Questions

**Comments to the Authors:**

**Please note here if the review is uploaded as an attachment.**

Reviewer #2: I would like to thank the authors for their careful revision of the manuscript. I have no further question. Congratulations!

Reviewer #3: The authors have done an excellent job in responding to my previous reviews. I have only one very minor points for them to address.

The type of wavelet as well as the wavelet parameters are not described in section S13

**********

**Have the authors made all data and (if applicable) computational code underlying the findings in their manuscript fully available?**

The PLOS Data policy requires authors to make all data and code underlying the findings described in their manuscript fully available without restriction, with rare exception (please refer to the Data Availability Statement in the manuscript PDF file). The data and code should be provided as part of the manuscript or its supporting information, or deposited to a public repository. For example, in addition to summary statistics, the data points behind means, medians and variance measures should be available. If there are restrictions on publicly sharing data or code —e.g. participant privacy or use of data from a third party—those must be specified.

Reviewer #2: **No:**  The authors indicate that the data will be available upon acceptance of the manuscript.

Reviewer #3: Yes

**********

PLOS authors have the option to publish the peer review history of their article (what does this mean?). If published, this will include your full peer review and any attached files.

If you choose “no”, your identity will remain anonymous but your review may still be made public.

**Do you want your identity to be public for this peer review?**  For information about this choice, including consent withdrawal, please see our Privacy Policy.

Reviewer #2: **Yes:**  Marine Vernet

Reviewer #3: **Yes:**  Avniel Singh Ghuman

# Editor report: Acceptance letter

**DOI:** 10.1371/journal.pcbi.1014563.r004

**Authors:** Daniele Marinazzo, Laura Dugué

PCOMPBIOL-D-25-01303R1

Single pulse electrical stimulation in white matter modulates iEEG visual responses in human early visual cortex

Dear Dr Huang,

I am pleased to inform you that your manuscript has been formally accepted for publication in PLOS Computational Biology. Your manuscript is now with our production department and you will be notified of the publication date in due course.

The corresponding author will soon be receiving a typeset proof for review, to ensure errors have not been introduced during production. Please review the PDF proof of your manuscript carefully, as this is the last chance to correct any errors. Please note that major changes, or those which affect the scientific understanding of the work, will likely cause delays to the publication date of your manuscript.

Soon after your final files are uploaded, unless you have opted out, the early version of your manuscript will be published online. The date of the early version will be your article's publication date. The final article will be published to the same URL, and all versions of the paper will be accessible to readers.

For Research, Software, and Methods articles, you will receive an invoice from PLOS for your publication fee after your manuscript has reached the completed accept phase. If you receive an email requesting payment before acceptance or for any other service, this may be a phishing scheme. Learn how to identify phishing emails and protect your accounts at https://explore.plos.org/phishing.

Thank you again for supporting PLOS Computational Biology and open-access publishing. We are looking forward to publishing your work!

With kind regards,

Sharmila Kamatchi

PLOS Computational Biology | Carlyle House, Carlyle Road, Cambridge CB4 3DN | United Kingdom ploscompbiol@plos.org | Phone +44 (0) 1223-442824 | ploscompbiol.org | @PLOSCompBiol
